# Membrane transporters for the special amino acid glutamine: structure/function relationships and relevance to human health

**DOI:** 10.3389/fchem.2014.00061

**Published:** 2014-08-11

**Authors:** Lorena Pochini, Mariafrancesca Scalise, Michele Galluccio, Cesare Indiveri

**Affiliations:** Department DiBEST (Biologia, Ecologia, Scienze della Terra) Unit of Biochemistry and Molecular Biotechnology, University of CalabriaArcavacata di Rende, Italy

**Keywords:** glutamine, amino acids, nutrients, membrane, transporters, cancer, homology models

## Abstract

Glutamine together with glucose is essential for body's homeostasis. It is the most abundant amino acid and is involved in many biosynthetic, regulatory and energy production processes. Several membrane transporters which differ in transport modes, ensure glutamine homeostasis by coordinating its absorption, reabsorption and delivery to tissues. These transporters belong to different protein families, are redundant and ubiquitous. Their classification, originally based on functional properties, has recently been associated with the SLC nomenclature. Function of glutamine transporters is studied in cells over-expressing the transporters or, more recently in proteoliposomes harboring the proteins extracted from animal tissues or over-expressed in microorganisms. The role of the glutamine transporters is linked to their transport modes and coupling with Na^+^ and H^+^. Most transporters share specificity for other neutral or cationic amino acids. Na^+^-dependent co-transporters efficiently accumulate glutamine while antiporters regulate the pools of glutamine and other amino acids. The most acknowledged glutamine transporters belong to the SLC1, 6, 7, and 38 families. The members involved in the homeostasis are the co-transporters B0AT1 and the SNAT members 1, 2, 3, 5, and 7; the antiporters ASCT2, LAT1 and 2. The last two are associated to the ancillary CD98 protein. Some information on regulation of the glutamine transporters exist, which, however, need to be deepened. No information at all is available on structures, besides some homology models obtained using similar bacterial transporters as templates. Some models of rat and human glutamine transporters highlight very similar structures between the orthologs. Moreover the presence of glycosylation and/or phosphorylation sites located at the extracellular or intracellular faces has been predicted. ASCT2 and LAT1 are over-expressed in several cancers, thus representing potential targets for pharmacological intervention.

## Introduction

**Figure 1:**
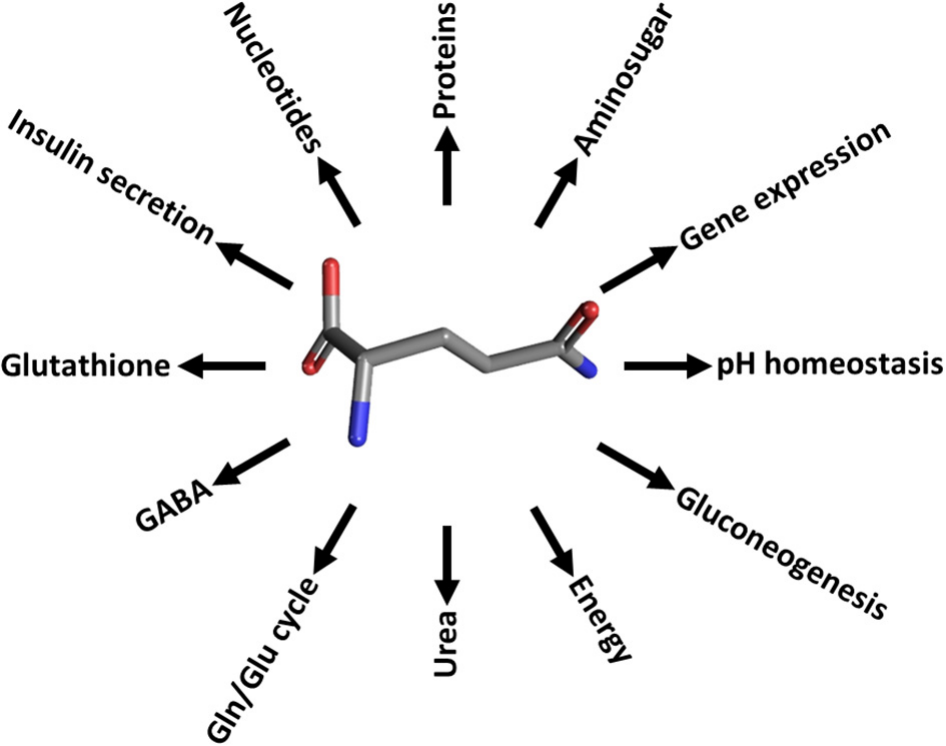
**The glutamine roles in cell pathways**. Schematic representation of the cell processes involving glutamine. Proteins, protein synthesis; Aminosugar, aminosugar synthesis; Nucleotides, purine and pyrimidine synthesis; pH homeostasis, mainteinance of acid-base balance; Gluconeogenesis, precursor synthesis; Energy, providing carbon atoms for TCA; Urea, release of NH_3_ in liver for urea synthesis; Gln/Glu cycle and GABA, neurotransmission regulation; Glutathione, GSH synthesis and redox balance regulation; Insulin secretion, glucose concentration regulation; Gene expression, gene expression regulation.

The pivotal role of glutamine in cell metabolism of mammals is well recognized. Besides glucose, glutamine represents a primary nutrient for maintenance of body's homeostasis (Bode, 2001; Newsholme et al., 2003; McGivan and Bungard, 2007). Therefore glutamine plasmatic concentration must be regulated and kept constant. Glutamine is the most abundant amino acid: the intracellular concentration ranges from 2 to 20 mM, while the extracellular one ranges from 0.2 to 0.8 mM (Bode, 2001; Newsholme et al., 2003). It is involved in many cell processes, the most important of which are depicted in Figure 1. Glutamine is precursor for proteins, amino sugar, purines and pyrimidines synthesis in different tissues; it is essential for acid-base buffering in kidney, being the most important donor of NH_3_ excreted in urine (Busque and Wagner, 2009; Verrey et al., 2009). Interestingly, in kidney and liver the carbon skeleton of glutamine enters the TCA cycle participating to gluconeogenesis. The amount of glucose produced through this pathway accounts for 25% of the circulating glucose which increases in case of diabetes (Stumvoll et al., 1999; Curi et al., 2005; Daye and Wellen, 2012). Glutamine represents fuel also for intestine, where it plays the additional role in maintaining gut integrity. Several reports show that glutamine administration helps recovery of intestinal integrity from pathological conditions (Ziegler et al., 2000). In liver glutamine provides nitrogen atoms for the urea cycle, a metabolic pathway compartmentalized between cytosol and mitochondria (Indiveri et al., 1994). In brain, besides a role as scavenger of NH_3_, glutamine participates to the glutamine/glutamate cycle, in which glutamine is synthesized from glutamate reabsorbed from synaptic cleft (Broer and Brookes, 2001; Bak et al., 2006; Conti and Melone, 2006). Glutamine is involved in the maintenance of redox potential balance in terms of GSH/GSSG ratio. GSH synthesis needs glycine, cysteine and glutamate, the latter deriving from glutamine through glutaminase action (Shanware et al., 2011; Daye and Wellen, 2012); moreover, glutamine entering the TCA cycle, gives rise to reducing equivalents, NADPH, used to increase GSH/GSSG ratio (Curi et al., 2005). Glutamine is linked to insulin secretion by pancreatic β–cells where it stimulates glucose oxidation with consequent increase of ATP/ADP ratio. This event induces membrane depolarization and results in increase of the intracellular Ca^2+^ level and, thus, insulin release (Newsholme et al., 2003). Lastly, an important role for glutamine is the regulation of several genes involved in signal transduction, metabolism, cell proliferation, cell defense and repair (Curi et al., 2005) and refs herein) (Figure 1). Taken together, these information highlight that glutamine has both acute and chronic effects on cell metabolism and functions. Therefore, glutamine must be considered “essential” even though it can be synthesized endogenously. Indeed, patients with critical illness (HIV infections, muscle disorders and side effects of chemotherapy) take advantage of glutamine supplementation. On the contrary, some concerns on glutamine-rich diet in healthy individual have been recently raised. Thus, further studies are needed to clarify these points (Holecek, 2013). The significance of glutamine for cell metabolism is proven by its role also in pathological conditions: cancer cells, in fact, have a special need for amino acids, such as glutamine, arginine and leucine which are those mainly required as reported in Section “Glutamine transporters in human pathology.” From the depicted scenario it emerges that the processes of glutamine absorption from diet, renal reabsorption and delivery to different tissues are essential and must be well coordinated. Therefore, different transport systems should work in a concerted manner to allow this special amino acid performing all its functions.

## Glutamine transporters: from functional to molecular classification

**Figure 2:**
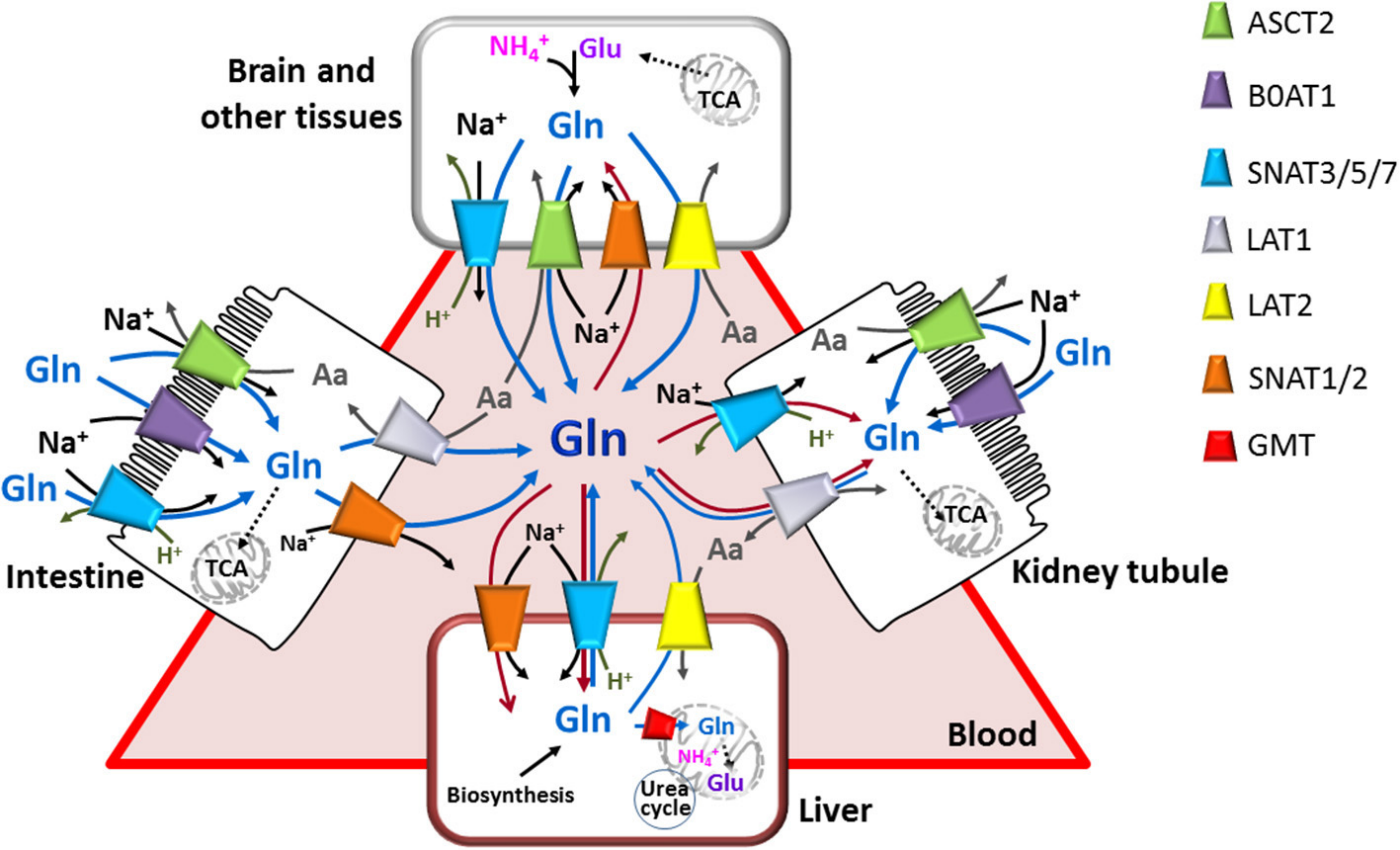
**The glutamine transporter network**. Interplay among epithelial polarized cells (apical membrane is depicted as brush-border; basolateral membrane is in contact with blood) and other cells. Glutamine transporters are indicated in the figure with different colors. Arrows indicate glutamine fluxes from (red) or toward (blue) blood or from lumen to epithelial cells (blue); black arrows indicate sodium fluxes; gray arrows indicate other amino acid and proton fluxes. Simplified cytosolic and mitochondrial pathways are depicted: synthesis of glutamine (in brain and other tissues), TCA, glutamine entering in TCA (intestine and kidney tubule), synthesis of glutamate from TCA intermediate (brain and other tissue) or from glutamine (liver), Urea cycle (liver).

Glutamine transport processes are guaranteed by a number of membrane transporters which share specificity for glutamine but show differences in transport modes. The pleiotropic role of glutamine may be the reason why glutamine transporters, belonging to several protein families, are redundant and ubiquitous (Figure 2). These proteins are, in general, specific for several neutral amino acids, therefore their classification, as well as their functional identification, were not unequivocal. The classification of these transporters was originally based on functional properties, such as substrate specificity, ion and pH dependence, kinetics and regulatory properties. This original classification led to cluster in “systems.” System A and System L were the first to be so-defined indicating “alanine-preferring” and “leucine-preferring,” respectively (Oxender and Christensen, 1963). The systems included one or even more transporters, still not identified as specific proteins, due to obvious difficulties to discriminate a single function in entire cells.

Over the years, a broad classification was adopted which differentiated between Na^+^-dependent and Na^+^-independent systems (Bode, 2001). Several transporters sharing the specificity for glutamine were described on the basis of specificity toward other amino acids, inhibitors and modes of transport such as System N which exhibits narrow specificity to glutamine, histidine and asparagine (Schioth et al., 2013), System ASC specific for alanine, serine and cysteine and System B0 with broad specificity to neutral amino acids. Among the Na^+^-dependent transporters, the best described are the transporters belonging to Systems A and N, the ASCT2 transporter which belongs to System ASC, B0AT1 belonging to system B0.

Among the Na^+^-independent transporters, LAT1 and LAT2 which belong to system L, are the best characterized. Some other less known transporters have been described whose relationship with glutamine homeostasis is, however, not unequivocal. These minor transporters will be dealt with to some extent in the sections below. A further feature used to distinguish different transporters was the high (such as systems N) or low (such as ASCT2) tolerance toward the substitution of Na^+^ by Li^+^. However, in 2003 Mackenzie et al suggested that Li^+^ tolerance is not *per se* a useful criterion to classify amino acid transporters, since in some instance it depends on membrane potential and on the parameters used in experiments (Mackenzie et al., 2003). Moreover, transporters can be characterized by the different specificity for amino acids and sensitivity to inhibitors; for example, the Systems A and L could be functionally identified by response to the amino acid analog MeAIB and BCH, respectively. Even though inhibitors allow to follow a transport reaction of a specific protein excluding that of other transporters, in the case of amino acid transporters, this criterion was not unequivocal due to the redundancy and the broad specificity of the glutamine transporters in cell.

Upon the identification of genes coding for amino acid transporters and the progress in wide genome sequencing, the classification of the glutamine transporters should be revised taking into account the sequence similarity and the presence of motifs corresponding to specific protein families. The SLC nomenclature may be helpful for a more structured clustering of the transporters. Several reviews already refer to SLC based classification for amino acid transporters (Mackenzie and Erickson, 2004; Hagglund et al., 2011; Bodoy et al., 2013; El-Gebali et al., 2013; Fotiadis et al., 2013; Kanai et al., 2013; Pramod et al., 2013; Rask-Andersen et al., 2013).

Referring to SLC, the classification of glutamine transporters is a little more complicated, since in some instances they are homologous to transporters for amino acids with different chemical properties. For these reasons, due to some discrepancies among similarity in function and similarity in amino acid sequences, the functional classification does not match completely the sequence similarity classification. SLC was previously used for the glutamine transporters by McGivan and Bungard together with the different names associated to the specific transporters (McGivan and Bungard, 2007). Table 1 resumes the two classifications for the glutamine transporters. The different modes of transport displayed by these transporters determine their roles in the different tissues and in polarized membranes (Figure 2). Thus, Na^+^-dependent symporters perform the function of efficiently absorbing glutamine in epithelia using the electrochemical gradient; this allows net accumulation of the amino acid against transmembrane gradient. Antiporters perform the function of regulating the balance between pools of glutamine and other amino acids or protons in specific tissues such as the neuronal subtypes; these transporters allow also glutamine efflux from specific tissues. Interaction of some of the glutamine transporters with accessory proteins has been described. In the case of the glutamine transporters LAT1 and LAT2, it seems that the accessory proteins are part of the transport competent complex or at least are important for stabilizing the protein into the plasma membrane (Costa et al., 2013; Rosell et al., 2014). We expect increasing evidences of interaction of the glutamine transporters with other proteins for regulative purposes. Regulation of the glutamine transporters is, indeed, essential for accomplishing its homeostasis, also in response to variations of intracellular and extracellular glutamine levels. However, relatively few information is available on this topic, so far.

**Table 1:** **The basic characteristics of the glutamine transporters**.

**Family**	**Member**	**System**	**Aliases**	**Mechanism**	**Variant**	**Regulator/ mode**
*SLC1*	*A5*	ASC	ASCT2, ATB0	Na^+^-glutamine/ neutral amino acids antiport	Isoform1: NP_005619.1	Glutamine/protein expression
					Isoform 2: NP_001138616.1	EGF/trafficking and activity
					Isoform 3: NP_001138617.1	Insulin andIGF/activity
						mTOR/protein expression
						Leptin/trafficking and gene expression
						pRb/protein expression
						Aldosterone/protein expression
*SLC6*	*A14*	B(^0,+^)	ATB^0,+^	2Na^+^-1Cl^−^-glutamine co-transport (electrogenic)	NP_009162.1	EGF and GH/expression
	*A19*	B or B^0^	B^0^AT1	Na^+^-glutamine co-transport, (electrogenic)	NP_001003841.1	collectrin (kidney), ACE2/trafficking
						APN (intestine)/activity and trafficking
						Leptin/trafficking and gene expression potassium/activity
						JAK2 (Janus kinase-2)/trafficking
						PKB-Akt and SGK/trafficking
	*A15*		B^0^AT2	Na^+^-glutamine co-transport, (electrogenic)	Isoform 1: NP_877499.1	
					Isoform 2: NP_060527.2	
					Isoform 3: NP_001139807.1	
*SLC7*	*A5*	L	LAT1	glutamine/ large neutral amino acids antiport	NP_003477.4	4F2hc/trafficking c-Myc/protein expression
					XP_006721350.1^*^	EAA/protein expression
					XP_006721349.1^*^	Glucose/up regulation Aldosterone /protein expression insulin/increases mRNA abundance
	*A8*		LAT2	glutamine/ small neutral amino acids antiport	Isoform 1: NP_036376.2	4F2hc/trafficking
					Isoform 2: NP_877392.1	Aldosterone/protein expression
					Isoform 3: NP_001253965.1	mTORC1/trafficking
					Isoform 4: NP_001253966.1	DHT/protein expression
	*A6*		y+LAT2	Na^+^-glutamine/cationic amino acids antiport	NP_001070253.1	4F2hc/ trafficking
*SLC38*	*A1*	A	SNAT1, ATA1, SAT1, NAT2	Na^+^-glutamine cotransport (electrogenic)	Isoform 1: NP_001265317.1	
					Isoform 2: NP_001265319.1	
	*A2*		SNAT2, ATA2, SAT2, SA1	Na^+^-glutamine cotransport (electrogenic)	Isoform 1: NP_061849.2	DHT/activity glucagon/expression
					Isoform 2: XP_005269040.1^*^Isoform 3: BAG57253.1^*^	
	*A3*	N	SNAT3, SN1, NAT	Na^+^-glutamine/H^+^antiport (electroneutral)	NP_006832.1	Insulin/trafficking
						PKC/trafficking
						Manganese/degradation
	*A5*		SNAT5, SN2	Na^+^-glutamine/H^+^ antiport (electroneutral)	Isoform 1: NP_277053.2	c-myc/expression
					Isoform 2: XP_005272752.1^*^	
	*A7*		SNAT7	Na^+^-glutamine/H^+^antiport (electroneutral)	Isoform 1: NP_060701.1	
					Isoform 2: XP_006721292.1^*^	

*The SLC and System classifications are reported; Mechanism of transport is described; variants reported in GeneBank are listed; known regulators and their effect are indicated*.

* *Predicted protein*.

**Figure 3:**
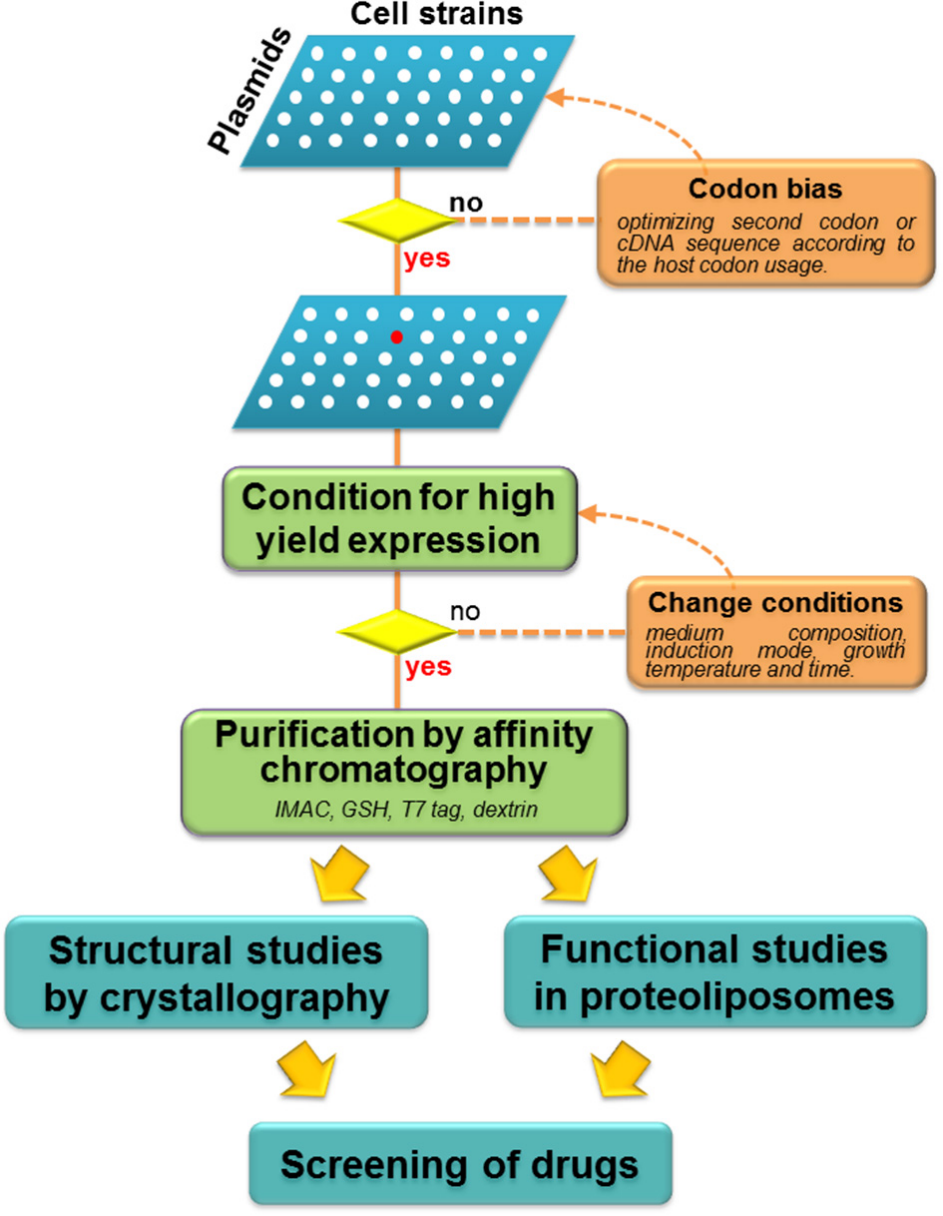
**Work flow of heterologous over-expression of membrane transporters**. Schematic representation of screening of different combination Plasmid/Cell strains (white spots): if the attempts with wild type gene is not successful, codon bias strategy should be applied. Thus, selection of the best plasmid/cell strain combination is performed (red spot) with optimization of conditions for high yield expression. When this result is achieved, purification procedures are applied to perform both structural and functional studies. These strategies allow large scale screening of potential drugs or xenobiotics.

Function of the glutamine transporters has been initially studied mainly in cell systems over-expressing the transporters. Significant advancements to the knowledge of the glutamine transporters derived from studies in artificial systems which give the important advantage to exclude interferences ascribed to other transporters and/or cell enzymes. In these systems, native transporters extracted from model animals were firstly reconstituted (Oppedisano et al., 2004; Oppedisano and Indiveri, 2008). The most promising approach is currently represented by the heterologous expression of the human isoforms of transporters in microorganisms. So far, three glutamine transporters have been successfully over-expressed in *E. coli* or in the yeast *P. pastoris*, interestingly, all at the same time (Costa et al., 2013; Galluccio et al., 2013; Pingitore et al., 2013), establishing the basic strategies (Figure 3) for further advancement of the knowledge on the glutamine transporter function and structure.

In the following paragraphs an update is given on the glutamine transporter knowledge concerning functional, regulatory and structural advancements and relationships with human health, respect to the last excellent comprehensive reviews on the glutamine transporters (Bode, 2001; McGivan and Bungard, 2007).

## SLC1A5: ASCT2

### Gene and tissue localization

*SLC1A5*, known as ASCT2, is a glutamine transporter belonging to the *SLC*1 family which encompasses five high affinity glutamate transporters (*SLC1A1, SLC1A2, SLC1A3, SLC1A6, SLC1A7*) and two neutral amino acid transporters (*SLC1A4* and *SLC1A5*). In humans, the five glutamate transporters possess 44–55% amino acid sequence identity with each other, whereas the two neutral amino acid transporters exhibit 57% identity. The human isoform of *SLC1A5* gene, isolated in 1996 from human placenta (Kekuda et al., 1996), is annotated on the chromosome 19q13.3. Three different transcripts exist in GeneBank, deriving from different translation start (Kanai et al., 2013); up to now the functional and kinetic characterizations have been conducted only on the first variant (NM_005628) constituted by 2873 nucleotides and 8 exons (Table 1). This transcript encodes a peptide of 541 amino acids. The dbSNP database reports more than 400 SNPs both in coding and non-coding region. Only the variant *SLC1A5-P17A* (rs3027956) is associated with breast cancer (Savas et al., 2006). *SLC1A5* gene is expressed in several tissues: kidney, intestine, brain, lung, skeletal muscle, placenta and pancreas (Kekuda et al., 1996; Utsunomiya-Tate et al., 1996; Broer and Brookes, 2001; Deitmer et al., 2003; Gliddon et al., 2009; Indiveri et al., 2014).

### Function

The acronym ASCT2 stands for AlaSerCys Transporter 2, even though the preferred substrate is glutamine. Indeed, the importance of ASCT2 for human health is linked to the ability to mediate delivery of this amino acid. The transport mechanism of this transporter has been widely studied in different experimental models: cell systems (Torres-Zamorano et al., 1998) as well as proteoliposomes reconstituted with the rat transporter extracted from kidney (Oppedisano et al., 2004, 2007; Pingitore et al., 2013). More recently, the human protein was over-expressed in *P. pastoris* and reconstituted in fully active form in proteoliposomes (Pingitore et al., 2013). This gave a further advancement to the knowledge of the human transporter. All the experimental approaches revealed similar basic properties of the transporter which works as a strictly Na^+^-dependent obligatory antiporter of neutral amino acids (Table 1). Na^+^ cannot be substituted by Li^+^. Experiments conducted with radioactive substrates as well as competition data confirmed that also alanine, threonine, serine, leucine, valine, asparagine, methionine, isoleucine, tryptophan, histidine, and phenylalanine are substrates of this transporter. Glutamate, lysine, arginine, MeAIB and BCH are not transported. By using proteoliposomes, important novelties on the human ASCT2 substrate specificity have been revealed. In particular, human ASCT2 showed an asymmetric specificity for amino acids. Glutamine, serine, asparagine, and threonine are bi-directionally transported while alanine, valine and methionine can be only inwardly transported. The functional asymmetry was also confirmed by kinetic analysis: different Km values were measured on the external and internal sides of proteoliposomes, 0.097 and 1.8 mM, respectively (Pingitore et al., 2013). The Na^+^_ex_ :amino acid_ex_ stoichiometry of transport is 1:1. The electrical aspect of the transport reaction is, however, not completely clarified: in fact, the inwardly-directed transport of Na^+^ should evoke electrical currents, leading to an electrogenic transport (Kekuda et al., 1996, 1997; Torres-Zamorano et al., 1998). Some groups, on the contrary, described an electroneutral transport (Utsunomiya-Tate et al., 1996; Broer et al., 1999). Interestingly, anion conductance activity has been described by electrophysiological measurements (Grewer and Grabsch, 2004). Very recently, using computational and experimental approaches a transport mechanism in which more than one Na^+^ ion is involved in amino acid translocation has been suggested. The study showed that even if the amino acid exchange occurs in an electroneutral mode, the transport cycle is indeed electrogenic due to the movement of Na^+^ ions across membrane. However, the used experimental model did not allow assay at the intracellular side (Zander et al., 2013). Thus, to gain further insights in the electrical properties of this transporter, a more appropriate model is needed, in which the intracellular environment might be better controlled; this would be useful also for defining the overall catalytic mechanism which is still underneath. The physiological role of hASCT2 has been extensively investigated in different tissue. ASCT2 in brain contributes to the glutamine-glutamate cycle (Figure 1) mediating the efflux of glutamine from astrocytes to recover the glutamate released in synaptic cleft (Broer et al., 1999). The same cycle occurs in placenta where glutamine enters the fetal liver to synthesize glutamate, which is then used for fetus metabolism (Torres-Zamorano et al., 1998). An evolutionary role has also been proposed for the human ASCT2 isoform: a group of retroviruses used this transporter as a receptor to infect human cells, giving rise to a co-evolution phenomenon (Marin et al., 2003). Moreover, among them, the human endogenous retroviruses (HERVs) have been detected as transcripts and proteins in the central nervous system. Associations among these retroviruses with syndromes including multiple sclerosis (MS) and several psychiatric disorders have been described. Binding of HERV proteins to ASCT-1 or -2 receptors might reduce the amino acid intake (Antony et al., 2011). One of the most important signaling function reported for ASCT2 relies on the link with the mTOR pathway. mTOR is an highly conserved Ser/Thr kinase that forms two complexes (mTORC1 and mTORC2) by interacting with several other proteins. mTOR pathway integrates signals from five major routes: growth factors, stress, energy status, oxygen and amino acids. In particular, leucine and arginine should be involved in activating mTOR. However, the link with the amino acid signals is still underneath. In this scenario, glutamine taken up by ASCT2 stimulates leucine uptake by a parallel leucine/glutamine antiport catalyzed by LAT1 (Nicklin et al., 2009).

### Regulatory aspects

Several data on regulation of ASCT2 expression is reported both in physiological and pathological conditions, even though the molecular mechanisms of regulation are mostly unknown (Table 1). One of the first report dealing with this issue showed that glutamine itself is able to regulate ASCT2 expression in human hepatoma cells (Bungard and McGivan, 2004). In the mentioned study, a region of 907 bp flanking the 5′ of ASCT2 gene has been cloned in the luciferase reporter system. In this system, glutamine strongly induces the promoter activity possibly via FXR/RXR dimer formation. This transcription factor complex binds to an IR-1 repeat of 24 bp in the promoter region of ASCT2. This has been further demonstrated by the site-directed mutagenesis of the IR-1 region. Furthermore, it has been postulated that the expression of FXR, which is linked to glucose availability, may positively regulate glutamine (Bungard and McGivan, 2005). Soon after, other groups proposed that glutamine transport through ASCT2 is stimulated by EGF signaling pathway (Palmada et al., 2005; Avissar et al., 2008). This should involve activation of PKC and protein kinase MEK. The uptake of amino acid is also regulated by insulin and IGF, which activate a cascade involving PI3K and the downstream targets SGK and PKB. The regulation of ASCT2 is due to increase of its abundance in plasma membrane even though ASCT2 does not harbor a canonical consensus sequence for these kinases (Palmada et al., 2005). This is in line with the observation that the transporter trafficking is a PI3K-dependent phenomenon and that the transporter stabilization in membrane is due to the GTPase Rho (Avissar et al., 2008). The link of ASCT2 with mTOR pathway has been suggested in 2007 by Fuchs et al.; the study showed that silencing ASCT2 in hepatoma cells causes reduction of mTOR activity, leading to apoptosis (Fuchs et al., 2007). Rapamycin decreases ASCT2 expression, indicating a reciprocal effect between this transporter and mTOR activity. The phenomenon underlying this mechanism is still underneath (Fuchs et al., 2007). Interestingly, in 2005 a study by Xu et al, reported the isolation of a super-complex constituted by LAT1/CD98, MCT/CD147 (lactate membrane transporter) heterodimers and ASCT2, which would be under regulation of AMPK and mTOR (Xu and Hemler, 2005). This would fit with the need of simultaneously regulating such transporters, which are fundamental for energetic metabolism, particularly in cancers (Ganapathy et al., 2009). Down-regulation of ASCT2 expression has been reported in intestine by the multifunction hormone leptin, which is involved in modulating the activity of several transporters of energy rich molecules (Ducroc et al., 2010). Lastly, the tumor suppressor pRb via E2F-3 transcription factor has been shown to regulate ASCT2 expression as well as other proteins involved in glutamine metabolism. Interestingly, in cancer the constitutive pRb degradation leads to continuous activation of E2F-3 and, then, enhanced expression of ASCT2 (Reynolds et al., 2014).

### Structural aspects

In spite of the different physiological roles in mammals, *SLC1* family members share common structural features. The tridimensional structure of the archeal homolog of the *SLC1* family, Gltph from *P. horikoshii*, was solved by X-ray crystallography (Yernool et al., 2004). The structure of Gltph has a trimeric architecture forming an aqueous cavity. In 2007 the same group solved the structure in presence of substrate and of a competitive inhibitor (Boudker et al., 2007), opening the possibility of docking analysis with substrates and inhibitors. Each protomer is an independent transport unit and is formed by eight transmembrane domains and two hairpin loops (HP1 and HP2) which are thought to be responsible of substrate translocation, being the mobile part of the transporter (Boudker et al., 2007; Reyes et al., 2009).

**Figure 4:**
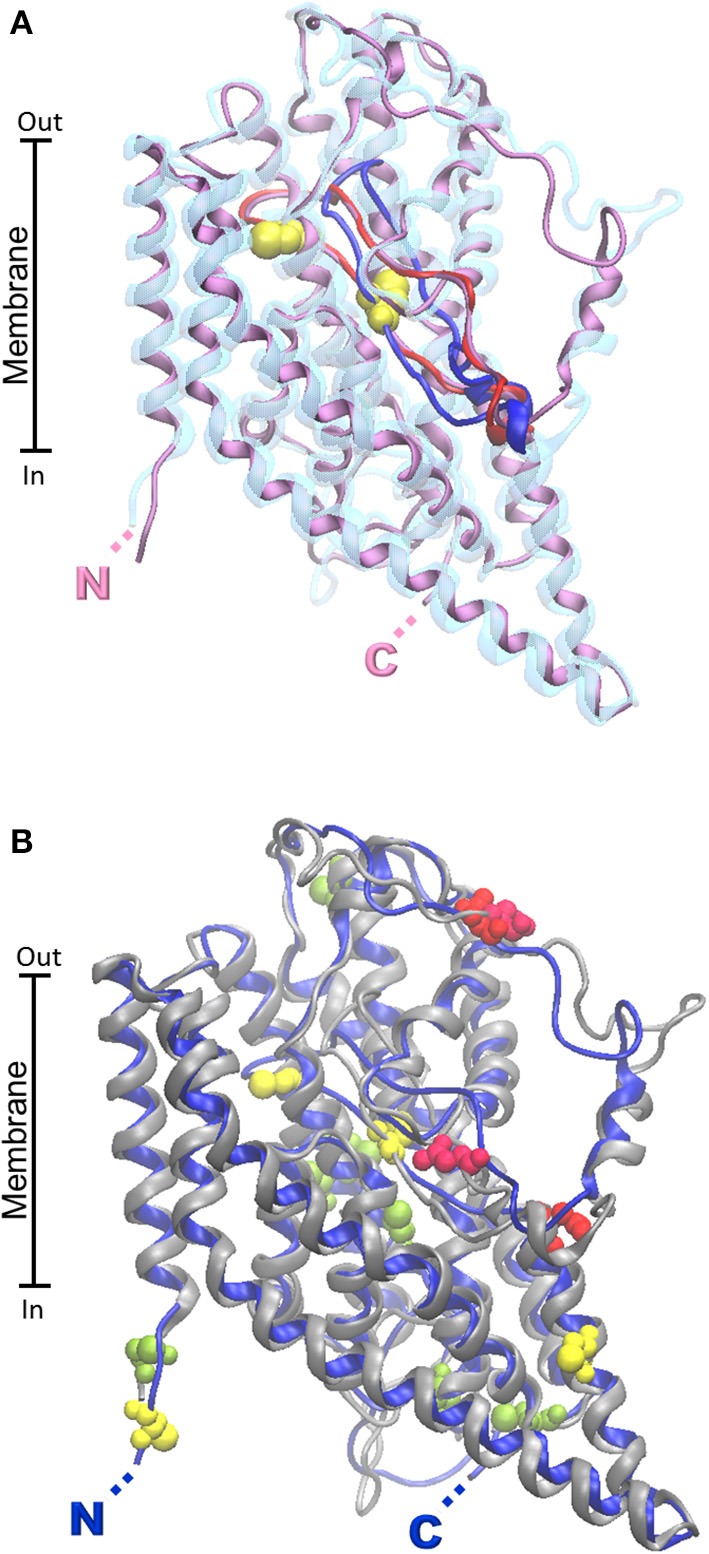
**Homology models of ASCT2 human and rat transporters**. The homology structural models of rat and human ASCT2 were obtained by the Modeler 9.13 software (Sali and Blundell, 1993) using as template the structure (PDB 1XFH) of the glutamate transporter homolog from *P. horikoshii* (Glpth). To run the software, sequences were aligned by ClustalX2 software with .pir output format. RMSD for model comparison was calculated by Spdbv 4.1.0. Superposition of the rat and human structural models was performed by VMD 1.9.1. **(A)** The human protein (transparent) contains a variable loop in bleu, the rat one (purple) contains a variable loop in red. The Cys residues of the CXXC metal binding motif present only in the rat protein are highlighted in yellow. **(B)** The human protein is in gray; the rat one is in bleu. Putative glycosylation sites of both proteins are highlighted in red. Cysteine residues common to the two orthologous proteins are highlighted in light green. Additional Cys residues present only in rat protein are highlighted in yellow. N- and C- terminals of rat and human proteins are nearly coincident and highlighted by single N and C.

The first homology model of rat ASCT2 has been constructed in 2010 (Oppedisano et al., 2010), followed by others which confirmed the putative structure of the glutamine transporter (Albers et al., 2012; Zander et al., 2013). More recently the human ortholog has been modeled (Pingitore et al., 2013). Homology models of human and rat ASCT2 are shown in Figure 4. Despite the 79% sequence identity between the two orthologous proteins, a very low identity degree (14%) has been found in local stretches (aa 200–229) as shown by the model (Figure 4A). The localization of this region is predicted in the vicinity of the substrate binding site (Oppedisano et al., 2010). Two putative glycosylation sites are reported in the homology model (N163, N212 for human and N164, N215 for rat) which are conserved and exposed toward the extracellular face, according with the proposed orientation of the protein in membrane (Figure 4B).

Another relevant difference between the two orthologs is the number of cysteines. 8 Cys residues are conserved in both the proteins, while the rat isoform contains additional 8 Cys residues. Two of these residues (C207 and C210) form, in the rat ASCT2, a CXXC metal binding motif missing in the human isoform. Function/structure relationships studied in proteoliposomes highlighted the importance of Cys residues in substrate translocation which is potently inhibited by specific Cys reagents. Interestingly, ASCT2 harbors the two hairpins, HP1 and HP2, which are important for substrate translocation (Pingitore et al., 2013). This finding suggests that the translocation mechanism may be similar to that of Glpth, even though ASCT2 works by an antiport mode of transport. Therefore, additional constraints are needed for coupling substrate transport in opposed directions.

## SLC6A19: B0AT1

### Gene and tissue localization

*SLC6A19* belongs to the *SLC6* also called Na^+^-dependent neurotransmitter transporter family in which only one out of 21 members is a well characterized glutamine transporter. Few other members of the family recognize glutamine but with lower affinity respect to other substrates (Verrey et al., 2005; Broer, 2006; Pramod et al., 2013).

B0AT1 was identified and cloned from mouse cDNA library; it shows all properties of the system previously defined as B0 (Broer et al., 2004). The transporter localization was found in proximal tubules and intestinal microvillus. B0AT1 was then recognized as one of the major transporters of neutral amino acids in these epithelia (Bohmer et al., 2005; Camargo et al., 2005; Broer et al., 2011) (Figure 2). Also in humans, B0AT1 has been localized in kidney and intestine. Lower, but significant expression has also been found in skin, pancreas, prostate, stomach, liver (Kleta et al., 2004; Seow et al., 2004). The genomic localization of the mouse B0AT1 is on chromosome 13 in cytoband C1, syntenic to human chromosome 5p15. The human gene is constituted by 12 exons. Two transcripts are reported in the data bases, but only one (5174 bp) codes for a protein, while the other one (3337 bp) undergoes nonsense mediated decay (Table 1).

### Function

The function of mouse and human B0AT1 was initially studied in oocytes. The transporter recognizes, besides glutamine, leucine, cysteine, valine, isoleucine, methionine, phenylalanine, alanine, serine, and asparagine as main substrates. Threonine, glycine, proline, histidine, tyrosine, tryptophan and BCH showed also some affinity for the transporter even though lower than glutamine. While arginine, lysine, aspartate, glutamate and MeAIB are not substrate. Half saturation constants of the mouse transporter for glutamine, measured by different authors, range from 0.5 mM to more than 3 mM (Broer et al., 2004; Bohmer et al., 2005). Km of the human transporter was reported to be 0.25 mM (Souba et al., 1992). However, the Km on the internal side of the transporter could not be measured in oocytes. Transport catalyzed by B0AT1 is well recognized to be electrogenic. An Hill coefficient of 1.5 of the Na^+^ dependence suggests that either two Na^+^ are co-transported with amino acids or that 1 Na^+^ is co-transported and a seconds second ion binds to an allosteric site (Broer et al., 2004). Further studies demonstrated a 1:1 stoichiometry (Bohmer et al., 2005; Camargo et al., 2005). The replacement of Na^+^ with Li^+^ is not tolerated. It was also suggested that the transporter may provide pathways for Na^+^, H^+^, and/or Cl^−^ that are not thermodynamically coupled to the amino acid transport indicating not saturable channel-like, as opposed to carrier-mediated activity (Camargo et al., 2005). This phenomenon is similar to that found for the mitochondrial carriers but has not a physiological explanation (Tonazzi and Indiveri, 2003) and refs herein). Regarding the mechanism of transport, either a random model was proposed (Bohmer et al., 2005) or an ordered mechanism (Camargo et al., 2005) in which Na^+^ would follow substrate binding. The discrepancies among the different studies are caused by differences in the experimental systems, by the confounding presence of multiple transport systems and by the complexity of the entire cell model (Camargo et al., 2005; McGivan and Bungard, 2007; Oppedisano and Indiveri, 2008). Significant clarification and advancement of the knowledge on B0AT1 were provided by the more recent studies of the protein extracted from rat kidney and inserted in proteoliposomes. In this experimental model, the transporter was inserted right side out respect to the cell orientation, thus giving the possibility to gain information without the interferences but ascribable to the cell context. Besides confirming some basic properties, such as substrate, ion specificity and inhibitor sensitivity, the artificial system gave information on the electrical nature of transport. Electrogenicity originates from Na^+^ transport coupled to glutamine, while Cl^−^ has no influence on the electrogenic behavior. Regulation by K^+^ was also found (see below). The half-saturation constant for glutamine in proteoliposomes corresponded to a Km measured in oocytes (Broer et al., 2004), i.e., 0.55 mM. It was clarified that the Na^+^-glutamine co-transport displays a 1:1 stoichiometry and the transport mechanism is random simultaneous. Interestingly, in proteoliposomes the intracellular Km could be measured. Its value is 2.0 mM indicating an asymmetric nature of the transporter (Oppedisano and Indiveri, 2008; Oppedisano et al., 2011). This data correlates well with the intracellular and extracellular glutamine concentrations. Indeed the concentration inside (about 2 mM) is much higher than that outside the cell (about 0.5 mM) (Cynober, 2002). These data allows to speculate that B0AT1 might, under some condition, catalyze also efflux of glutamine (Oppedisano et al., 2011).

It can be concluded that the absorption of glutamine and other neutral amino acids from intestine and reabsorption in kidney are the primary functions of this transporter (Figure 2).

### Regulatory aspects

Study on regulation of B0AT1 is at an advanced stage (Table 1). The carboxypeptidase ACE2 and collectrin (non-peptidase homolog of ACE2) have been described as key regulators of intestinal and renal amino acid uptake (Fairweather et al., 2012). Interestingly, collectrin was shown to specifically increase B0AT1 activity probably by enhancing its surface expression (Danilczyk et al., 2006), not the intrinsic transport function. Proteoliposome experiments demonstrated that collectrin has no essential role in the catalysis of glutamine transport by B0AT1 (Oppedisano et al., 2011). B0AT1 forms complexes with APN that increases the affinity of the transporter for substrate up to 2.5-fold besides increasing its surface expression (Fairweather et al., 2012). Leptin modulates intestinal glutamine absorption through reduction of B0AT1 trafficking to the plasma membrane (Ducroc et al., 2010).

Allosteric regulation by K^+^ has been described. K^+^ exerts a biphasic modulation of the transporter interacting at the intracellular site. Up to 50 mM internal (intracellular) K^+^, transport was stimulated while at higher K^+^ level it was inhibited. Since the intracellular K^+^ level depends on ATP concentration, it can be assessed that the nucleotide indirectly modulates the B0AT1 transporter activity. High ATP level, causing increase in K^+^, would impair B0AT1 activity; lower ATP level will stimulate B0AT1 (Oppedisano and Indiveri, 2008). This regulation is opposed to the regulation by ATP found for the rat ASCT2.

The Janus Kinase 2 enhances the protein abundance in the cell membrane (Bhavsar et al., 2011). PKB/Akt up-regulates *SLC6A19* activity, which may foster amino acid uptake into PKB/Akt-expressing epithelial and tumor cells (Bogatikov et al., 2012).

### Structural aspects

According to protein similarity, B0AT1 displays LeuT fold. The main properties influencing the substrate affinity are: the presence of the charged amino and carboxyl groups, L-configuration (not D-) of the α-carbon, net neutral charge, size and, to a lesser extent, hydrophobicity of the side-chains (Yamashita et al., 2005). Uncharged amino acids are favored due to the predicted interaction with a hydrophobic pocket. B0AT1 has similar substrate specificity of LeuT with the exception of tryptophan, that is weakly transported only by B0AT1 (Camargo et al., 2005; Yamashita et al., 2005; Broer, 2006; O'mara et al., 2006). Another difference is the co-transport of 2 Na^+^ in the case of LeuT, while only 1 Na^+^ is co-transported by B0AT1. The role of the seconds second Na^+^-binding site in B0AT1 remains unclear. The homology model highlighted a structural asymmetry which correlates with the function and explains the structural basis of the loss of function of some mutants (Broer, 2008, 2009).

**Figure 5:**
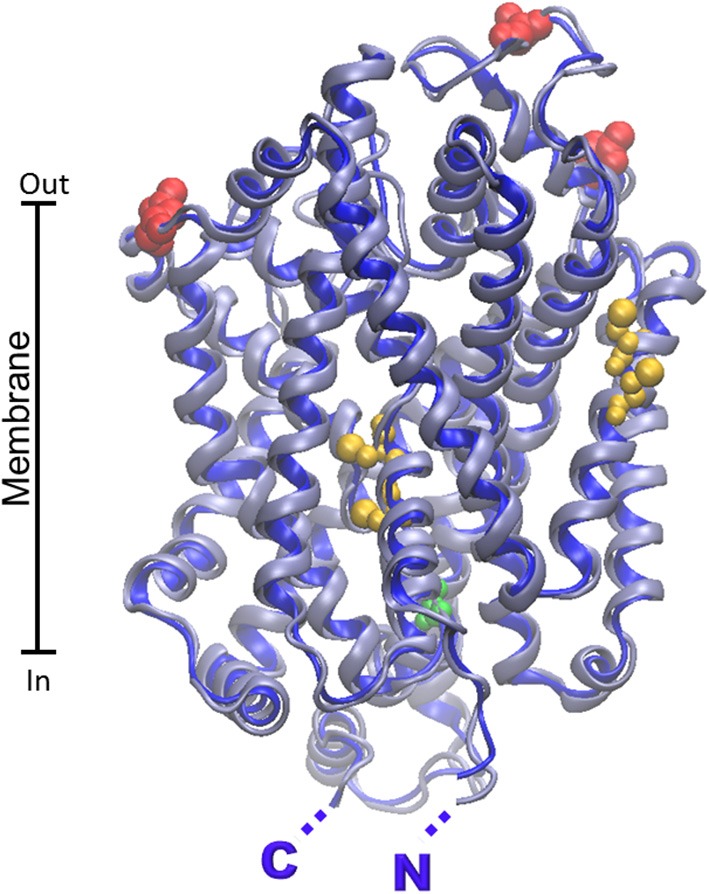
**Homology models of B0AT1 human and rat transporters**. The homology structural models of rat (gray) and human (blue) B0AT1 were constructed as described in Figure 4 using as template the structure of dopamine transporter from *D. melanogaster* (PDB 4M48). RMSD for model comparison was calculated by Spdbv 4.1.0. Superposition of the rat and human structural models was performed by VMD 1.9.1. Putative glycosylation sites are highlighted in red; cysteine residues of the metal binding motifs are highlighted in yellow; PKC phosphorylation site is highlighted in green. N- and C- terminals of rat and human proteins are nearly coincident and highlighted by single N and C.

More recently, the dopamine transporter from *Drosophila melanogaster* (PDB 4M48) has been crystallized and resolved at 2.95 Å (Penmatsa et al., 2013). The alignment of the B0AT1 with the dopamine transporter has higher identity (33%) respect to the LeuT transporter (18%), thus a more accurate modeling could be obtained. The superposition of the rat and human structural models (0.43 Å RMSD) shows that the two orthologs are very similar (Figure 5), as expected from the 87% identity between the human and rat protein and differently from ASCT2 (Figure 4A). This similarity correlates well to the similar functional properties of the two orthologs.

Database analysis (ExPASy PROSITE, http://www.expasy.ch/prosite) of regulatory sites within the SLC6A19 amino acid sequence predicted five *N*-glycosylation sites. Three (N158, N182, N258) are reported in the homology model (Figure 5). The others are not present since the region 332–377 containing these residues cannot be modeled. According to the predicted orientation, the glycosylation sites protrude toward the extracellular side. The SLC6A19 sequence contains also a conserved intracellular consensus site (S100) for phosphorylation (Figure 5) which is known to regulate a variety of channels and transporters (Bohmer et al., 2010). Moreover, Figure 5 highlights the presence of two conserved metal binding motifs in the middle of the transporter, the CXXC (containing C200/C203) and the CXXXC motifs (containing C45/C49). The first is located close to the membrane face, far from the hypothetical substrate binding site; the seconds second is in the core of the protein, closer to the substrate binding pocket. The metal binding sites correlate well with the interaction of heavy metals with the transporter, as described below (Oppedisano et al., 2011).

## SLC6A14: ATB0^,+^

*SLC6A14* is the molecular counterpart of ATB^0,+^ belonging to the system B^0+^. The hATB0^,+^ was the first cloned amino acid transporter of system B^0+^ (Sloan and Mager, 1999). It is expressed in colon, lung (Sloan et al., 2003), eye (Ganapathy and Ganapathy, 2005) and mammary gland (Nakanishi et al., 2001) (Table 1). This is a broad specificity transporter which recognizes glutamine with relatively low affinity (Bode, 2001) Km 0.6 mM (Sloan and Mager, 1999) and other neutral as well as cationic amino acids but also carnitine and carnitine derivatives. Therefore, it can be considered a minor glutamine transporter. Transport catalyzed by ATB^0,+^ is dependent on Na^+^ and Cl^−^ transmembrane gradients and is sensitive to membrane potential. 2 Na^+^ and 1 Cl^−^ are involved in the transport (Nakanishi et al., 2001). As found for ASCT2, ATB0^,+^ may be up-regulated in cancer to meet the increasing demand of arginine (Gupta et al., 2005, 2006), representing a potential target of cancer therapy (Karunakaran et al., 2011). In this sense the selective blocker methyl-DL-tryptophan (α-MT) or the BCH acid might represent an important chemical scaffold for drug design (Sloan and Mager, 1999).

## SLC6A15: B0AT2

In 2006 it was demonstrated that the mouse v7-3 gene encodes a transporter for neutral amino acids named B0AT2, due to the high similarity with B0AT1 (Broer, 2006) (Table 1). The results indicated that B0AT2 mediates Na^+^-dependent uptake of leucine, isoleucine, valine, proline, methionine and with a very low affinity glutamine. The transport is electrogenic with a stoichiometry of 1 Na^+^:1 amino acid. Besides kidney, it is highly expressed in cerebellum and brain. This localization indicates that B0AT2 might be mainly involved in providing neurotransmitter precursors to neurons. Nitrogen from leucine, in fact, is transferred to oxoglutarate to form glutamate; isoleucine, methionine and valine can be metabolized to succynil-CoA for glutamate biosynthesis. Thus, B0AT2 has marginal if any role in glutamine homeostasis.

## Glutamine transporters of the SLC7 family

Important glutamine transporters belong to the *SLC7* family which accounts for 13 members divided in two subgroups: the Cationic Amino acid Transporters (CATs) and the Light subunits of Amino acid Transporters (LATs) of the Heterodimeric Amino acid Transporters. Some general information will be given here to highlight the peculiarity of the glutamine transporters belonging to this family. HATs and CATs originate from a common ancestor with 12 trans-membrane domains (TMD) which duplicated the last two TMD originating a 14 TMD structure (Verrey et al., 2004; Hansen et al., 2011; Fotiadis et al., 2013). Only the HATs group includes glutamine transporters. They mediate obligatory antiport of a broad spectrum of substrates (Fotiadis et al., 2013). HATs emerged in metazoan and represent one of the few example of transporters composed by two different subunits. The Light subunit of HATs, LATs, are known as glycoprotein-associated amino acid transporters since they associate with heavy subunit belonging to the SLC3 family via a conserved disulphide bridge (Broer and Brookes, 2001; Wagner et al., 2001; Palacin and Kanai, 2004). This is a very small family of type II (with C-ter outside the cells) membrane glycoproteins comprising SLC3A1 and SLC3A2, named rBAT and 4F2hc, respectively. The main function of SLC3 proteins consists in routing the transporters to plasma membrane forming the HAT complex. 4F2hc (heavy chain of the cell surface antigen 4F2) is a multifunctional protein involved, besides transport, in immuno system regulation, cell activation, growth and adhesion (Palacin and Kanai, 2004; Cantor and Ginsberg, 2012; Fotiadis et al., 2013). Furthermore, 4F2hc has been suggested to play a role in integrin signaling and activation linking this protein to human malignancies (Cantor and Ginsberg, 2012).

So far, six human light subunits have been identified as partners of 4F2hc: LAT1, LAT2, y+LAT1, y+LAT2, asc1 and xCT all belonging to the *SLC7* family. This broad spectrum of interaction justifies the ubiquitous expression of 4F2hc and the absence of pathology linked to its complete loss which is, indeed, not compatible with life (Palacin and Kanai, 2004). Interestingly, an heterodimer complex formed with the glucose transporter GLUT1 has been reported, confirming the regulatory role of 4F2hc in general transport mechanism (Ohno et al., 2011). As stated above, the main interaction between LATs and heavy chain occurs via disulphide formation; this, however, is not essential since mutation of Cys does not abolish completely the presence of HATs in membrane (Pfeiffer et al., 1998; Broer et al., 2001; Palacin and Kanai, 2004; Franca et al., 2005; Rosell et al., 2014). The following paragraph deals with the members of SLC7 involved in glutamine disposition.

## SLC7A5: LAT1

### Gene and tissue localization

The rat and human cDNAs encoding LAT1 protein have been isolated and identified in 1998 and confirmed in 1999 (Kanai et al., 1998; Prasad et al., 1999). The human gene has been annotated in the chromosome 16q24.3 and is characterized by 10 exons. Three transcripts are reported on ensemble database, but only one protein has been characterized. The longest (4537 bp) codes for a protein of 507 amino acid. Other two isoforms of 241 and 334 amino acids are also predicted (Table 1), but without evidence of transport activity. LAT1 is expressed in brain, ovary, testis, placenta, spleen, colon, blood-brain barrier, fetal liver, activated lymphocytes, skeletal muscle, heart, lung, thymus, and kidney (Kanai et al., 1998; Yoon et al., 2005). It is mainly localized in the basolateral membranes of polarized epithelia (Verrey et al., 2004; Fotiadis et al., 2013) (Figure 2).

### Function

The first report describing hLAT1 activity (Prasad et al., 1999) conducted in cell systems, revealed that LAT1 works as heterodimer. In several studies it has been assessed that LAT1 mediates obligatory antiport of tryptophan, phenylalanine, leucine and histidine with higher affinity (Km in humans ranging from 5 to 50 μM) (Del Amo et al., 2008), glutamine and threonine with lower affinity; alanine, proline and charged amino acids are not recognized as substrates. The non-metabolizable analog BCH is a transportable inhibitor of LAT1(Mastroberardino et al., 1998). Interestingly, LAT1/4F2hc activity has been linked also to transport of L-dopamine across the blood-brain barrier suggesting a potential role of LAT1 in Parkinson's disease (Kageyama et al., 2000). Transport of thyroid hormones by LAT1 was also described being important in neurological development (Kinne et al., 2011). The covalent interaction between LAT1 and 4F2hc requires the conserved C164 of human LAT1(C 165 in rat) and the conserved C109 of human 4F2hc (C103 in rat) (Fort et al., 2007); either the N-ter and the extracellular parts of 4F2hc are essential for non-covalent interaction as demonstrated by experiments with truncated mutants (Broer et al., 2001; Franca et al., 2005). The transport mode has been described as Na^+^ and pH independent (Table 1); this allows the relative concentration of large neutral amino acids acting synergistically with other Na^+^-coupled amino acid transporters, such as ASCT2 or unidirectional transporters such as system A and N (Verrey, 2003; Del Amo et al., 2008). LAT1 is also involved in regulation of signaling via mTOR, which is responsible of cell growth and survival. This feature shed light on the link between LAT1 and cancer (see Section “Glutamine transporters in human pathology”). In OMIM databank no pathologies are associated to inherited mutations of LAT1 gene. Further information on unknown functional aspects of human isoform of LAT1 will come from the availability of the large scale over-expressed protein (Galluccio et al., 2013). The achievement of such objective represents a great challenge in protein field since amino acid transporters are known to be toxic for bacteria (Miroux and Walker, 1996). hLAT1 represents, in fact, the only case of a mammalian amino acid transporter expressed in bacteria (Galluccio et al., 2013). A strategy previously used for OCTN subfamily was adopted for LAT1and 4F2hc over-expression (Indiveri et al., 2013) (Figure 3). After screening of plasmid and cell combinations, the pH6EX3 plasmid revealed suitable for significant over-expression of the protein in *E. coli* Rosetta DE3pLysS (Brizio et al., 2006; Torchetti et al., 2011). Optimization of some parameters, such as temperature growth and IPTG concentrations (Brizio et al., 2006) was performed and followed by an affinity chromatography procedures (Galluccio et al., 2013). The importance of this study relies on the unique possibility of handling the human protein alone or in combination with the accessory protein 4F2hc (CD98) to characterize function and kinetics.

## SLC7A8: LAT2

### Gene and tissue localization

LAT2 gene from human and murine source was reported in 1999 by different groups (Pineda et al., 1999; Segawa et al., 1999). The human gene is located in the chromosome 14q11.2 and encodes for a protein of 535 amino acids with 12 putative TMDs. Human LAT2 is mainly expressed in kidney, placenta, brain and at lower extent in spleen, skeletal muscle, small intestine, lung (Pineda et al., 1999; Fraga et al., 2005; Yoon et al., 2005; Fotiadis et al., 2013) but also in prostate, ovaries, testis and fetal liver (Park et al., 2005). As in the case of LAT1, LAT2 is mainly localized at the basolateral membrane of polarized epithelia cells (Figure 2). Alignment with hLAT1 shows 50% identity. Alternative transcripts are annotated in GeneBank coding for three additional but uncharacterized proteins (Table 1).

### Function

In cell systems, it has been shown that only the co-expression of hLAT2 and 4F2hc mediates uptake of neutral amino acids which is highly trans-stimulated and Na^+^-independent. LAT2/4F2hc is inhibited by the amino acid analog BCH as LAT1. The main difference with LAT1 is in the substrate choice: while LAT1 prefers large neutral amino acids, LAT2 has a broader specificity mediating transport of small neutral amino acids such as alanine, glycine and serine as well. Glutamine, for which LAT1 has low affinity, is one of the main substrates of LAT2. However, LAT2 shows a general lower affinity for substrates respect to LAT1, with Km ranging in humans from 0.2 to 1 mM (Del Amo et al., 2008). Differently from LAT1, LAT2 is sensitive to more acidic pH (Christensen, 1990). The expression of LAT2 in brain, liver and skeletal muscle suggests a role in mediating release of glutamine. An important advance in LAT2 functional knowledge came from the successful over-expression in *P. pastoris* of the heterodimer 4F2hc/LAT2 (Costa et al., 2013). Soon after the structural bases of the interaction between the two subunits were revealed. This was confirmed by reconstitution in proteoliposomes of the heterodimer. These experiments indicated that 4F2hc is required for LAT2 stabilization in membrane but not for modulating substrate affinity (Rosell et al., 2014).

### Regulatory aspects of LAT1 and LAT2 heterodimers

The role played by LAT1 and LAT2 consists in equilibrating glutamine and other amino acids pool into the cells, while their net uptake derives from other transporters (Del Amo et al., 2008). The study on the regulation of LAT1/4F2hc is of great importance due to the key role played by this protein in several human cancers (See Section “Glutamine transporters in human pathology”). One of the first reports concerning this issue has been conducted in lymphocytes, where the basal expression of LAT1/4F2hc has been found increased upon activation signals. Studying the promoter regions of the two genes by 5′ RACE analysis and Run on assay suggested that control of LAT1 relates to transcriptional activity not to the half-life of mRNA (Nii et al., 2001). More recently, the LAT1 promoter has been analyzed in human pancreatic cancer cells in which LAT1 plays key function in promoting cell growth. The study revealed that LAT1 promoter harbors a canonical binding sequence for the proto-oncogene c-myc (Hayashi et al., 2012). This protein is an important transcription factor whose expression is often altered in cancers since it regulates expression of several genes controlling metabolism and cell cycle (Daye and Wellen, 2012). Silencing c-myc by siRNA leads to down-regulation of LAT1 expression in prostate cancer cells with subsequent decrease of proliferation, due to impairment of neutral amino acids uptake. Mutations of c-myc binding sequence on LAT1 promoter causes loss of responsiveness to c-myc expression. However, the mechanism of control c-myc-mediated may be more complicated since this protein can exert both epigenetic control and can regulate mRNA translation efficiency by microRNA expression (Hayashi et al., 2012). A recent study described that ingestion of essential amino acids resulted in humans in a transient increase of LAT1/4F2hc heterodimer; this event has been linked to activation of mTORC1 signaling and protein synthesis. The augmented expression might be an adaptive response to long term amino acid availability and/or to anabolic stimulus (Drummond et al., 2010). In line with this, a recent finding shows that embryo of null mice for LAT1 gene has a phenotype incompatible with life (Poncet et al., 2014). Glucose deprivation, due to pathological conditions such ischemia, has been also linked to up-regulation of LAT1 via cis-activation of an E-box on its promoter region in retina (Matsuyama et al., 2012). Low insulin concentration increases *SLC7A5*/LAT1 mRNA abundance in an mTORC1-dependent manner in skeletal muscle cells (Walker et al., 2014). Lastly, LAT1 protein expression has been shown to be increased upon chronic treatment with aldosterone (Amaral et al., 2008). Much less is known about regulation of hLAT2 that responds like LAT1 to aldosterone stimulus (Amaral et al., 2008). A regulation of LAT2/4F2hc expression by mTORC1 has been described in glomerular epithelial cells: in glomerulonephritis conditions, mTORC1 integrates signals from inflammatory cytokines stimulating LAT2 translocation to plasma membrane (Kurayama et al., 2011). Increased surface LAT2 expression by DHT, via EGF receptor involving the ERK1/2 cascade, has been described (Hamdi and Mutungi, 2011). The information above reported are summarized in Table 1.

### Structural aspects of LAT1 and LAT2

HATs seem to consist of a light chain (*SLC7A5–11*) with 12 putative TMD with the N- and C-terminals localized intracellularly and an heavy chain with the C-terminal localized extracellularly (Verrey et al., 2004; Costa et al., 2013). As stated above, the 12 TMD of the light chains show considerable similarity to the first 12 TMD of the CAT transporters. The heavy chain 4F2hc is a N-glycosylated protein (4 putative N-glycosylation sites: N264,280,323,405) with one transmembrane domain. The structure of the extracellular domain of human 4F2hc has been solved by X-ray diffraction (Fort et al., 2007). On the contrary, the structure of human LATs is not known, but Cys-scanning mutagenesis showed that the Cys residue between TMD III and IV is conserved being responsible of inter-subunit disulfide bridge with the heavy chain. LATs show significant similarity to the arginine/agmantine antiporter AdiC, to the broad-specificity amino acid transporter ApcT and to the glutamate/GABA antiporter from *E. coli* whose structures have been solved by X-ray crystallography (Shaffer et al., 2009; Ma et al., 2012). A recent study confirmed this hypothesis for LAT1 suggesting a translocation mechanism in which the role of Na^+^ is mimicked by a proton with an alternating transport mechanism (Forrest et al., 2011; Geier et al., 2013). The availability of this model allowed screening and molecular docking of potential anti-tumor drugs, revealing new substrates for LAT1 (Geier et al., 2013). Structural studies on LAT2 have been conducted upon over-expression of the heterodimer in *P. pastoris* with subsequent large scale purification (Costa et al., 2013; Rosell et al., 2014). The study shows, by different experimental approaches, that the extracellular domain of 4F2hc covers the surface of human LAT2 modeled on the basis of AdiC transporter. The docking model fitted the steric hindrance derived by cross-linking experiments and highlighted that the Cys residue involved in the formation of disulfide is the C154 between TMD III and IV. Interestingly, the interaction mode described shed new light on the role of 4F2hc on the path cycle of LAT2: the strong interaction of the heavy chain with a specific domain, named “hash domain,” of LAT2 makes this part of the heterodimer the more static (close to TMD 11 and 12), while the “boundle domain” including helices 1, 2, 6, 7, and loops 7–8 is the one moving during the transport cycle opening and closing the transporter (Rosell et al., 2014). This mechanism might be shared also by the other HATs.

**Figure 6:**
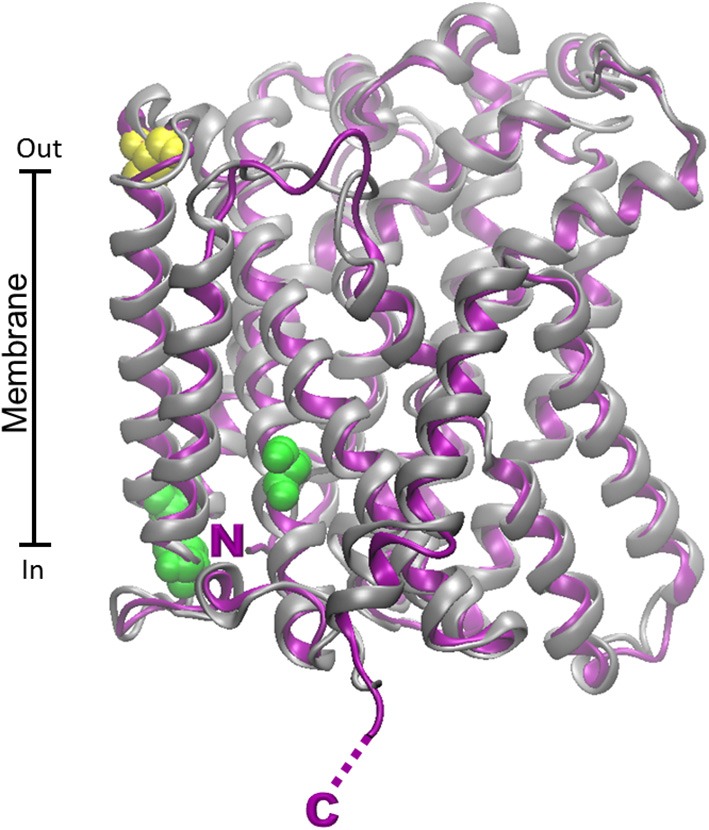
**Homology models of LAT2 human and rat transporters**. The homology structural models of rat (Chaudhry et al.) and human (purple) LAT2 were constructed as described in Figure 4 using as template the structure of the arginine/agmantine antiporter AdiC from *E. coli* (PDB 3OB6). Superposition of the rat and human structural models was performed by VMD 1.9.1. C154 of the human protein involved in disulfide bridge with 4F2hc is highlighted in yellow; putative PKC and PKA phosphorylation sites are highlighted in green. N- and C- terminals of rat and human proteins are nearly coincident and highlighted by single N and C.

Further structural features were already described for LAT1 and LAT2 transporters: potential casein kinase II-dependent phosphorylation site(s), PKC phosphorylation motifs, tyrosine kinase dependent phosphorylation sites (Prasad et al., 1999; Segawa et al., 1999). The alignment of the rat and human LAT2 sequences shows high percentage of identity (92%, not shown). This data has been confirmed by the superimposition of the two structural model (RMSD 0.66) obtained by using the structure of ADiC transporter as template (PDB 3OB6; Figure 6). Differently from the other glutamine transporters, Scan-Prosite analysis predicts lack of glycosylation sites. For these transporters the targeting to the membrane is guaranteed by their binding partner (Costa et al., 2013; Rosell et al., 2014). This interaction is mediated by disulfide bridge involving C154 for the human isoform and C155 for the rat isoform exposed toward the extracellular side of the proteins (Figure 6). Both the isoforms contain 11 Cys residues. No metal binding motif has been found. On the opposite side the human model contains 3 serine residues (S179, S337, S487), putative PKC phosphorylation sites and a threonine residue (T363), putative PKA phosphorylation site. These sites are exposed toward the cytosolic side of the protein. The rat and human proteins show nearly overlapping predicted structures (Figure 6).

## SLC7A6: y+LAT2

Together with LAT1 and LAT2, this transporter belongs to the *SLC7* family. The gene encoding the human isoform of y+LAT2 has been isolated in 1999 and annotated on the chromosome 16q22.1 (Pfeiffer et al., 1999). The protein is constituted by 515 amino acids and is mainly expressed in brain, small intestine, testis, parotids, heart, kidney, lung. y+LAT2 works as heterodimer with 4F2hc like the other members of *SLC7* family. It has been identified as arginine/glutamine exchanger: this role would be particularly important in brain where the protein might supply cells with important amino acids. Very interestingly, the catalytic mechanism of this transporter is unique: y+LAT2, indeed, mediates uptake of neutral amino acids in a Na^+^-dependent fashion, while cationic amino acid transport is Na^+^-independent (Table 1). The exchange occurs with a 1:1 stoichiometry. The antiport mechanism is expected to be electroneutral because the positive charge of the cationic amino acid is compensated for the Na^+^ ion accompanying the neutral amino acid (Broer et al., 2000).

## SLC7A15: ArpAT

The gene encoding this transporter has been annotated by *in silico* approach. BLAST analysis identified on mouse genome a region containing a putative ORF fulfilling the requirements of a transporter belonging to *SLC7* family: it has 64% similarity with b^0,+^AT, 12 TMD domains, 2 putative N-glycosylation sites present also in LAT1 and the conserved Cys residue involved in disulfide with *SLC3* member. This mouse transporter was then cloned and functionally characterized in HeLa cells; its over-expression in the presence of rBAT or 4F2hc increased neutral amino acid uptake. The transporter mediates uptake of alanine, serine, glutamine and cystine in Na^+^-and pH-independent manner. Analogously to LATs, ArpAT showed trans-stimulation. This transporter is expressed in intestine and, at much lower levels, in brain. The main physiological role played by ArpAT may be linked to providing dopamine to the non-neuronal dopaminergic system which regulates intestinal Na^+^ absorption (Fernandez et al., 2005). The most intriguing finding concerning this transporter is its evolutionary history: arpAT gene is, in fact, conserved in rat, dog, and chicken while it has been silenced in humans and chimpanzee. This suggests that its role became, along with primates evolution, not essential. The group which identified the transporter speculated that this may find explanation in the different diet of primates compared to other vertebrates (Fernandez et al., 2005).

## SLC38: SNATs

### Genes and tissue localization

The *SLC38* family belongs to the Amino acid Polyamine-organo Cation family (APC family) and includes 11 membrane transporters most of which are known to mediate the net uptake of glutamine, alanine, asparagine, histidine, arginine, and some other neutral amino acids in those tissues where they are expressed (Hagglund et al., 2011; Schioth et al., 2013). The most acknowledged transporters from this family are 6 proteins, originally classified as systems A and N and subsequently as SNATs or SATs and SNs, respectively. In this more recent classification, the numbers assigned to SNATs (SAT or SN) correspond to the identification numbers of the *SLC38* members. The other five members are still at early stage of characterization. SNAT1, 2, and 4 (*SLC38A1, 2* and *4*) correspond to the old systems A. System A was firstly isolated in 1965 (Christensen et al., 1965) and was defined by the inhibition with the amino acid analog MeAIB. In 2000 the three transporters belonging to such family were identified and the coding genes annotated on chromosome 12. The proteins SNAT1 and SNAT2 are 486 and 505 amino acids in length, respectively. The system N includes SNAT3, 5, and 7 (*SLC38A3, 5* and *7*). SNAT3 gene was identified in 1999 and annotated on the chromosome 3p21.31 (Chaudhry et al., 1999) encoding a protein of 503 amino acids. Then, SNAT5 gene was cloned in 2001 and annotated on the chromosome Xp11.23 (Nakanishi et al., 2001) encoding a protein of 471 amino acids. SNAT7 gene is localized on chromosome 16q21 and encodes a protein of 461 amino acids. According to GeneBank, different transcripts exist for these three genes, reported in Table 1. The tissue distribution of SNATs is wide and specific for each member: SNAT1 and SNAT2 are ubiquitous (Albers et al., 2001; Chaudhry et al., 2002); SNAT3 is expressed in liver, skeletal muscle, kidney and pancreas (Chaudhry et al., 1999); SNAT 5 is expressed in stomach, brain, liver, lung, small intestine, spleen, colon and kidney (Nakanishi et al., 2001); SNAT7 is ubiquitous (Hagglund et al., 2011). Regarding sub-cellular localization, SNATs are mostly localized in the basolateral membrane of absorptive epithelia (Schioth et al., 2013; Broer, 2014) (Figure 2). SNAT1 seems to be also localized in intracellular organelles in GABAergic neurons (Solbu et al., 2010). In nervous system, SNAT1 and SNAT2 are expressed in GABAergic and glutamatergic neurons (Solbu et al., 2010) while SNAT3 and SNAT5 are expressed in astrocytes (Chaudhry et al., 2002). In pancreas SNAT2 is localized in the α-cell membranes while SNAT3 in the β-cell ones. In kidney SNAT3 is localized on the basolateral membranes of the S3 segment of proximal tubules. In intestine an apical sub-cellular localization has been proposed for SNAT5. *SLC38* family comprises also other five additional members whose function is still orphan. *SLC38A6* (SNAT6) gene is localized on the chromosome 14q23.1 and is expressed mainly in brain and liver (Nakanishi et al., 2001). *SLC38A8* (SNAT8) gene is localized on the chromosome 16q23.3 and the protein has a distribution similar to that of SNAT7. The transcripts of the other members *SLC38A9, SLC38A10* and *SLC38A11* derive from genes annotated on chromosome 5q11.1, 17q25.3 and 2q24.3 respectively (Sundberg et al., 2008).

### Function

SNAT1 and 2 show a broad substrate specificity with preference for glutamine besides methionine, proline, serine, asparagine, glycine and histidine, while SNAT 4 is not considered as glutamine transporter (Schioth et al., 2013; Broer, 2014) and refs herein). SNAT 7, which was characterized later than SNAT 3 and 5, recognizes also arginine as substrate. SNAT7, considered member of system N, shows also characteristics of system A, such as broad substrate specificity (Hagglund et al., 2011). The known members of the *SLC38* family work as co-transporters for Na^+^, in which amino acid uptake is driven by the Na^+^ electrochemical gradient, inwardly directed (Mackenzie and Erickson, 2004) (Table 1). For system A members (SNAT1, 2) the uptake of glutamine results to be electrogenic, since each transport cycle causes a net movement of a positive charge with a stoichiometry 1:1. On the contrary, the transporters belonging to system N are also able to counter transport Na^+^ and H^+^ giving rise to an electroneutral transport mechanism. SNAT3 and 5, but not SNAT7, have a unique hallmark to tolerate substitution of Na^+^ with Li^+^ (Kilberg and Christensen, 1980; Mackenzie et al., 2003; Hagglund et al., 2011). All the known SLC38 transporters are activated by increasing pH from 6 to 8 (Mackenzie and Erickson, 2004). In addition to Na^+^ or H^+^ coupled transport, SNAT3 mediates uncoupled ion movement across the membrane (Schneider et al., 2007). Thus, protons and other cations can pass through the transporter by substrate dependent mode, while other ion conductances are substrate independent. Whether this uncoupled transport has a physiological function it is still unclear as above described for B^0^AT1. Interestingly, a single residue, N76, is critical for coupled and uncoupled ion flows in the glutamine transporter SNAT3 (Broer, 2009). Moreover, it has been reported that a mutation of residue T380 affects the ability of Li^+^ to replace Na^+^ (Chaudhry et al., 2001).

Under a physiological point of view, SNATs play different role according to their localization (Figure 2). In nervous tissue, members of system a take up glutamine released by members of system N giving rise to the glutamine/glutamate cycle in neurons. In astrocytes, glutamine is synthesized via glutamine synthetase, using glutamate released by synapses and taken up via EAAT1 (Glutamate transporter *SLC1A3*) or EAAT2 (Glutamate transporter *SLC1A2*) (Schioth et al., 2013). Another example of concerted action among SNATs occurs in pancreas among alpha and beta-cells. At low plasmatic glutamine concentrations, SNAT3 releases glutamine supplying substrate for SNAT2, stimulating glucagon secretion. In the opposite condition, in the presence of high glutamine concentrations, differently by what above described, SNAT3 will accumulate glutamine which is then converted to glutamate. This reaction provides intermediate for TCA cycle, producing ATP that, similarly to what occurs in the presence of glucose accumulated in the β-cells via GLUT2, will inhibit KC potassium channels causing depolarization. This event facilitates fusion of insulin containing secretory granules with the plasma membrane (Jenstad and Chaudhry, 2013). Thus, SNAT3 which catalyzes both glutamine uptake and efflux can be considered a sensor of the nutritional state of the cell. However, glutamine affinity at the intracellular face is still not known, due to the difficulty in accessing the intracellular space in experiments performed with intact cells. In kidney SNAT3 contributes to glutamine absorption from the circulation; this is involved in regulation of acid-base homeostasis (Busque and Wagner, 2009). In liver SNAT1, SNAT2, SNAT3, and SNAT5 contribute to net import of amino acids, particularly alanine and glutamine regulating gluconeogenesis and giving rise to the glutamine/alanine cycle between liver and muscle (Kondou et al., 2013). In intestine, where glutamine uptake from lumen is mediated by B^0^AT1 (Figure 2), transporters from system A have been proposed as the main responsible of glutamine transfer to blood (Broer, 2008). Interestingly, in this body district SNAT5 would be involved in glutamine metabolism in crypts cells, even though further investigations are needed (Saha et al., 2012).

The functional and kinetic characterizations described in the cited papers, have been conducted in cell systems; so far, no example of over-expression and reconstitution in proteoliposomes of SNAT members is available. Thus, given their complex interplay, some aspects are still unknown.

### Regulatory aspects

The regulation of SNAT expression is a complex issue, not completely solved; the available information have been included in Table 1. SNAT1 expression is stimulated by PKA (Ogura et al., 2007). For SNAT2, a regulation by amino acid deprivation has been described and the mechanism linked to the presence in the first intron of a amino acid response element (AARE) acting as enhancer (Palii et al., 2004). Moreover, in liver SNAT2 is up-regulated by glucagon (Ortiz et al., 2011). For SNAT3 a regulation by PKC has been reported and linked to the glutamate/GABA-glutamine of the central nervous system. Derangement of SNAT3 regulation may cause pathological states both in the central nervous system and in peripheral organs (Nissen-Meyer and Chaudhry, 2013). Moreover, SNAT5 expression has been found regulated by c-myc in cancer cells (Deberardinis et al., 2007).

### Structural aspects

**Figure 7:**
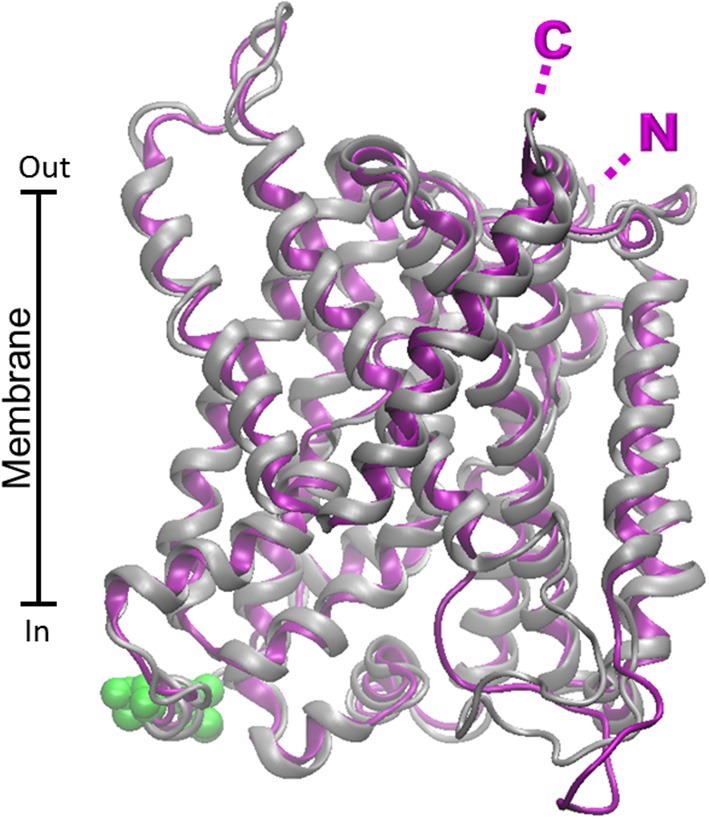
**Homology models of SNAT7 human and rat transporters**. The homology structural models of rat (purple) and human (Chaudhry et al.) SNAT7 were constructed as described in Figure 6. Superposition of the rat and human structural models was performed by VMD 1.9.1. Putative PKC and PKA phosphorylation sites are highlighted in green. N- and C- terminals of rat and human proteins are nearly coincident and highlighted by single N and C.

The structure of the *SLC38* members is characterized by a 5+5 inverted repeat fold typical of the bacterial LeuT (Broer, 2014). Recently, by bioinformatics and labeling experiments, the homology model of SNAT4 (built on LeuT and AdiC) has been validated (Shi et al., 2011). In this model the N- and C-terminals of the protein are extracellular, as well as three loops containing two N-glycosylation sites (N260 and N264). Interestingly, a disulphide bridge linking C249 and C321 is essential for SNAT4 function, which, however, is not a glutamine transporter. (Padmanabhan Iyer et al., 2013). The structural model of SNAT7 has been built as a representative of the *SLC38* glutamine transporters, given that it was the last characterized and, hence, an homology model was not yet built (Figure 7). Interestingly, the alignment of SNAT7 with the other *SLC38* members shows the lowest percentage of identity from 19 to 21% (not shown). In addition, SNAT7 has unique properties resembling those of both system A and N. Differently by *SLC1* and *SLC6* transporters, homology models built for human and rat SNAT7 proteins by Modeler do not have the same fidelity of the others due to the low sequence similarity to the available crystallized bacterial homologs (AdiC and ApcT). SNAT2 model was already built on the basis of LeuT crystal structure (Zhang et al., 2009). SNAT7 has the same topology of the homology model previously described for SNAT4 (Shi et al., 2011) with extracellular C- and N- terminals. Differently by SNAT4 no N-glycosylation sites have been predicted by bioinformatics for the human and rat SNAT7 proteins. Concerning the phosphorylation sites, a PKC binding site (T174) and a PKA binding site (T179) have been found in the human isoform. The same phosphorylation sites have been found in rat shifted of one residue.

## The mitochondrial glutamine transporter

A mystery in the glutamine transporter ensemble is still represented by the mitochondrial member. This transporter should be essential in metabolism since release of ammonia by glutamine deriving from extra hepatic tissues, should occur inside the mitochondrial matrix, where the ubiquitous form of glutaminase, i.e., derived from GLS1 gene, is localized (Figure 2). Glutaminase deriving from GLS1 gene exists in two splicing variants, both localized in mitochondria (Katt and Cerione, 2014). Interestingly and in line with the role of glutamine in cancer cell metabolism (see Section “Glutamine transporters in human pathology”), the GLS1expression is altered in cancer. This has been linked with suppression of miRNA23a and b via both c-myc and NFkB pathways (Gao et al., 2009; Rathore et al., 2012). Indeed, the metabolic flux of ammonia has been well demonstrated to finish in mitochondria also on the basis of a recent work on pancreatic cancer cells, in which glutamine transfer to mitochondria, via transporters, is hypothesized due to the impairment of the cell growth when GLS1 is knocked down (Son et al., 2013). In spite of the great importance of the mitochondrial glutamine transporter, only preliminary evidences on this transport function were provided by old studies in intact mitochondria (Sastrasinh and Sastrasinh, 1989) and then the protein responsible for this function was isolated and purified from mitochondrial extracts (Indiveri et al., 1998). Afterwards no other advances on the identification of the protein and/or the gene encoding the mitochondrial glutamine transporter have been achieved. Interestingly, the mentioned glutamine transporter has a higher apparent molecular mass, 41.5 kDa, than canonical mitochondrial carriers, i.e., 28–34 kDa. Therefore, the mitochondrial glutamine transporter may or not belong to the mitochondrial carrier family. Indeed, a mitochondrial carrier with a longer N-terminal extension has been described (Palmieri, 2008) as well as a mitochondrial transporter which does not belong to the mitochondrial carrier family (Herzig et al., 2012).

## Glutamine transporters in human pathology

**Figure 8:**
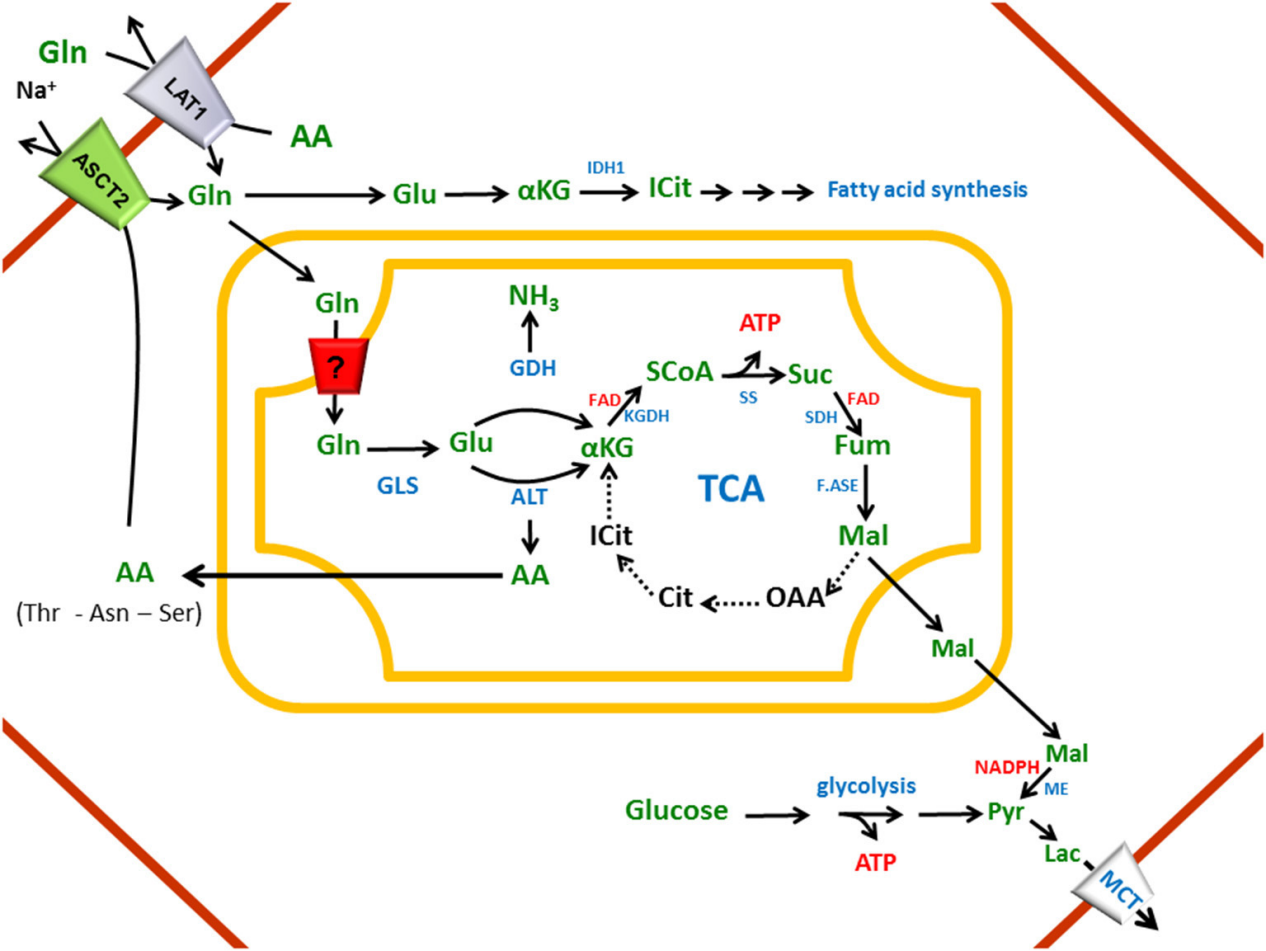
**Network of transporters involved in cancer metabolic switch**. In cell membrane (red), ASCT2 and LAT1: glutamine plasma membrane transporters; MCT: Monocarboxylate Transporter. In cytosol (upper part of the figure), Gln: glutamine, Glu: glutamate, αKG: α Ketoglutarate, ICIT: isocitrate, IDH1: Isocitrate dehydrognase 1 and simplification of reactions to fatty acid synthesis; (lower part of the figure) Mal: Malate, Lac: lactate and simplification of glycolysis with the end product pyruvate (Pyr). In the Inner Mitochondrial Membrane, putative glutamine transporter (?). In mitochondrial matrix, TCA (Tricarboxylic Acid Cycle) with enzymes, GLS: Glutaminase, GDH: Glutamate dehyfrogenase, ALT: Alanine Amino Transferase. AA: Amino Acid. Dotted arrows indicate metabolic pathways depressed in cancers.

The involvement of glutamine transporters in human pathology has been investigated and, with the only exception of the autosomal recessive disorder Hartnup disease, the only pathology linked to alteration of glutamine homeostasis, is cancer. Glutamine plays pivotal role in cell proliferation which requires nutrients, energy and increased biosynthetic activity leading to overall metabolic changes. In this scenario, cells shift to high glycolysis rate, lactate production and increased lipid biosynthesis. These pathways have been firstly described in lymphocytes which represent an useful model for studying cell proliferation (Deberardinis et al., 2008; Vander Heiden, 2011). Interestingly, a similar metabolic shift is a typical feature of cancer cells that need glucose and glutamine to sustain the high rate of proliferation (Fuchs and Bode, 2005; Ganapathy et al., 2009; Chen and Russo, 2012; Daye and Wellen, 2012; Son et al., 2013). Differential expression of specific genes, in particular those coding for plasma membrane transporters, allows increased uptake of the two nutrients. Normal proliferating cells need to integrate signals coming from extracellular growth factors, while cancer cells have increased autonomy. The main metabolic changes occurring in cancer are collectively known as Warburg effect (Ganapathy et al., 2009; Vander Heiden et al., 2009; Dang, 2010; Ohh, 2012). This phenomenon relies, besides increased glucose utilization, also on glutamine, canonically classified as “non-essential” amino acid that gives rise to a truncated form of TCA, terminating with malate and producing ATP at the substrate level (Figure 8). Malate is converted to pyruvate and lactate allowing cancer cells to produce ATP and NADPH necessary for anabolic processes (Figure 8). Lactate is exported by MCTs which are also over-expressed in tumors (Halestrap, 2013). Moreover, glutamine sustains cytosolic citrate synthesis via IDH1 and lipogenesis (Metallo, 2012) (Figure 8).

Therefore, it is not surprising that plasma membrane transporters responsible of glutamine disposition are altered in several human cancers. Among the transporters described in the above Sections, LAT1 (*SLC7A5*) and ASCT2 (*SLC1A5*) are those for which over expression in several tumors has been documented since 2005 (Fuchs and Bode, 2005). Specific examples of human cancers are listed in Table 2. In nearly all cases, over-expression of ASCT2 and LAT1 correlates with high grade of malignancies of these tumors. However, the role of ASCT2 in providing glutamine to cells (Nicklin et al., 2009) must be revisited in view of the well assessed obligatory antiport and functional asymmetry (Pingitore et al., 2013). Energy supply from glutamine will not result from the entire glutamine molecule (five carbon atoms) but from the difference between the carbon atoms taken up as glutamine and those exported as smaller amino acids such as serine and threonine (Figure 8). This difference accounts for one or two carbon atoms which are oxidized in the truncated TCA. The membrane transporters SNAT1, SNAT3, and SNAT 5 have been also shown over-expressed in some cancers (Table 2), to accomplish glutamine supply; these findings opened perspectives on SNATs as pharmacological targets, even though lack of suitable experimental tool made this issue still difficult to approach. Noteworthy, the low affinity glutamine transporter ATB^0,+^ is also up-regulated in some tumors, even though this is linked with its primary function of providing arginine to cells (Gupta et al., 2005).

**Table 2:** **SNATs, ASCT2, and LAT1 associated cancers**.

**Cancer type**	***SLC38* (SNATs)**	***SLC1A5* (ASCT2)**	***SLC7A5* (LAT1)**
Prostate cancer	Okudaira et al., 2011	Busque et al., 2009; Li et al., 2013	Patel et al., 2013; Segawa et al., 2013
Colorectal cancer		Witte et al., 2002	Ebara et al., 2010
Hepato Cell Carcinoma	Kondoh et al., 2007	Bode et al., 2002; Fuchs et al., 2004	Li et al., 2013
Lung cancer		Hassanein et al., 2013; Shimizu et al., 2014	Kaira et al., 2010; Imai et al., 2010
Breast cancer	Wang et al., 2013	Kim et al., 2012	Furuya et al., 2012
Neuroblastoma and glioma	Sidoryk et al., 2004	Wasa et al., 2002; Dolinska et al., 2003	
Endometrioid carcinoma			Watanabe et al., 2014
Ovarian Cancer			Kaji et al., 2010
Renal Cell Carcinoma			Betsunoh et al., 2013
Pancreatic and biliary tract cancer			Yanagisawa et al., 2012; Kaira et al., 2013
Gastric cancer			Shi et al., 2013
Pleural Mesothelioma			Kaira et al., 2011

*List of cancer tissues in which SNATs, ASCT2 and/or LAT1 have been found over-expressed with related references*.

Only one inherited pathology, named Hartnup disorder (OMIM 234500), is clearly associated to a glutamine transporter, i.e., B^0^ AT1. So far, 21 mutations have been described as causative of such pathology onset. This metabolic syndrome is complex and characterized by pellagra-like rash, cerebellar ataxia, emotional instability and strong aminoaciduria. Even though B^0^ AT1 is responsible also of glutamine absorption and reabsorption, the amino acids for which underlies its role in Hartnup disease are mainly histidine and tryptophan (Broer, 2009). The dramatic loss of such amino acids in urine is responsible of decreased serotonin and melatonin syntheses, explaining the neurological and skin problems, respectively.

## Toxicology and pharmacology: ASCT2 and LAT1 and drug interactions

The results above described highlighted the importance of glutamine membrane transporters in metabolic changes for cancer development and/or progression. This shed light, since years, on the pharmacological relevance of ASCT2 and LAT1 which became hot targets of drug discovery. Moreover, the pivotal role played by plasma membrane transporters in interaction with drugs is becoming more and more important and several evidences link these proteins to drug disposition among tissues and among different subcellular compartments (Giacomini et al., 2010; Pochini et al., 2013; Scalise et al., 2013). The challenge of advancing in this research field is represented by the possibility of designing good inhibitors able to specifically target the protein of interest. These studies started since some years and different groups reported attempts of designing and identifying specific inhibitors of ASCT2 (Grewer and Grabsch, 2004; Albers et al., 2012) and LAT1 (Geier et al., 2013). The design of specific and selective molecules requires the knowledge of the tridimensional structures of the target. However, so far, nearly no structures of human secondary membrane transporters have been solved due to the difficulty in handling them and to the lack of methodologies for over-expressing and folding. Only very recently the human isoform of GLUT1 transporter has been solved by X-ray diffraction, even though in an inactive form obtained by site-directed mutagenesis (Deng et al., 2014). The possibility to identify new compounds is therefore mostly based on integration between experimental procedures and bioinformatics, through homology modeling and molecular docking. The homology model structures previously obtained (Oppedisano et al., 2010, 2011; Pingitore et al., 2013; Pochini et al., 2014) and now improved (Figures 4, 5) have been used for several toxicological studies (see below). In the meantime, a lot of efforts have been made to obtain human isoforms of ASCT2 and LAT1 by over-expressing them in heterologous systems, following the work flow depicted in Figure 3. These procedures are useful to recover high yield of purified protein needed for both functional and structural studies. The two amino acid transporters hASCT2 and hLAT1/CD98 have been recently cloned and over-expressed in the yeast *P. pastoris* and in the bacterium *E. coli*, respectively (Galluccio et al., 2013; Pingitore et al., 2013). Even though the two proteins are both glutamine transporters, a common strategy could not be applied. This was not surprising given the experience already acquired with the three members of OCTN family (Indiveri et al., 2013).

The availability of these proteins in purified and in native form opens the possibility of obtaining X-ray structure for better defining or validating the already available homology models depicted in Figures 4, 6. Combining the over-expression systems and the procedures of proteoliposomes reconstitution, it will be soon possible to develop assays for high throughput screening of chemical compounds for drug discovery and design before animal experimentation (Pochini et al., 2013; Scalise et al., 2013), as depicted in the work flow of Figure 3. These kind of studies can be performed also in cell systems stably or transiently over-expressing the protein of interest; however, given the pleiotropy of glutamine and the broad substrate specificity of amino acid transporters, a strategy allowing the study of isolated proteins in artificial membranes is indispensable as suggested by several authors (McGivan and Bungard, 2007; Scalise et al., 2013; Schioth et al., 2013). By mean of proteoliposome reconstitution, some studies evaluating toxicological aspects of glutamine transporters have been already performed on the rat protein, extracted in native form from kidney, being the human isoforms not available at that time (see above in ASCT2 and B0AT1 Sections). Such studies gave the possibility of confirming also structure/function relationships speculated on the basis of homology models presented in Figures 4, 5. The works moved from the observation that some amino acid residues, such as Cys, increased their relative concentrations during protein evolution (Jordan et al., 2005). This makes Cys a potential target of different SH-reactive molecules; noteworthy the glutamine transporters dealt with the present review harbor several Cys residues. ASCT2 and B0AT1, indeed, were shown to be inhibited by several SH-reagents. Under a toxicological point of view, an interesting class of SH-reagents are the mercurial compounds. These molecules are potent environmental contaminants coming from industries with high toxicity for humans and in general for mammals since they enter the food chain as bio-product of bacterial metabolism (Rooney, 2007). A first work has been performed with ASCT2; this protein showed to be potently inhibited by HgCl_2_, methyl-mercury and other heavy metals (Oppedisano et al., 2010). The compounds interact very probably with a CXXC metal binding motif located at the end of the large hydrophilic loop of the transporter in a basin surrounded by hydrophobic residues (Figure 4A). The IC50 measured for mercury compounds are in the range of toxicity threshold. These results explain at the molecular level the toxic effect of methyl-mercury in brain (Boado et al., 2005). A large scale screening of drugs has been performed on ASCT2 in the same system (Oppedisano et al., 2012). In particular, potential anti-tumor drugs, dithiazoles, where designed specifically for rat ASCT2 and tested to identify the structural features of the most potent ones; the study revealed again that the CXXC motif is involved in covalent interaction with those drugs.

Another study in the proteoliposomes system has been conducted on B0AT1 extracted from rat kidney. Even though the structure of rat B0AT1 is not related with that of rat ASCT2, the transporters share the common feature of carrying CXXC metal binding motifs. In the case of B0AT1, the CXXC motif has been localized far from the putative binding site, correlating with the non-competitive inhibition by mercury compounds (Oppedisano et al., 2011) (Figure 5). B0AT1 contains, in addition, a CXXXC motif which has been described previously as target of metals such as Cu^2+^, Pb^2+^, Hg^2+^, Cd^2+^, and Zn^2+^ (Abajian and Rosenzweig, 2006; Tonazzi and Indiveri, 2011). This seconds second motif might explain the effect exerted by the prototype of mercury agent, mersalyl. In this case, in fact, substrate protection of the inhibition was found (Oppedisano et al., 2011). Interestingly, anti-oxidants are able to reverse the inhibition in both ASCT2 and B0AT1 proteins (Scalise et al., 2013). Very recently, a large scale screening of drugs has been performed on rat B0AT1 describing a specific inhibitory effect exerted by the common anti-inflammatory drug nimesulide (Pochini et al., 2014). Experimental data were supported by molecular docking of nimesulide on the rat B0AT1 showing that the site of drug interaction corresponds to that for antidepressants in SERT and GABA transporters (Krishnamurthy and Gouaux, 2012; Penmatsa et al., 2013). This feature, together with the high degree of identity between rat and human, makes reconstituted B0AT1 an important model for pharmacological and toxicological screening with applicability in humans. On the contrary, among the glutamine transporters described in this review, ASCT2 has the unique feature of being locally different between rat and human orthologs, making the availability of the human over-expressed transporter essential for applications in human health.

## Conclusion

Glutamine is a “conditionally essential” amino acid which homeostasis is maintained owing to the different mode of transport performed by several membrane transporters. Thus, on the one hand, concentrative transporters allow glutamine absorption from diet in intestine and reabsorption from urine in kidney. On the other hand, both co-transporters and antiporters allow delivery of glutamine in all the tissues and equilibration with other amino acids. The redundancy of transporters with overlapping functional properties is very probably related to the need of avoiding derangements from homeostasis even in case of malfunctioning of one transporter. The lack of known defects of genes encoding these transporters, with very few exception, is in some instances an evidence that derangements of glutamine homeostasis is incompatible with life. Notwithstanding all the transporters dealt with share specificity for glutamine, they display different tridimensional structures. This is in line with their classification in different protein families; however, within the same family, the orthologs are very conserved from rat to human. The only exception is represented by ASCT2 (*SLC1A5*) which may be related with some specific signaling role of the transporter arisen later in evolution. While the function of several transporters has been widely described, no structures are available and the molecular mechanisms of regulation are still not completely solved. Thus, a lot remains to be done for a comprehensive knowledge of glutamine fluxes through tissues. This is very important not only for the biochemical knowledge of the glutamine homeostasis but also, and more importantly, for the involvement of this special amino acid in the hottest topic of human health: cancer biology.

## Author contributions

Lorena Pochini, Mariafrancesca Scalise contributed in collecting bibliography, preparing figures and writing; Michele Galluccio contributed in drawing structure figures and writing; Cesare Indiveri contributed in writing and supervision of all the activities.

### Conflict of interest statement

The authors declare that the research was conducted in the absence of any commercial or financial relationships that could be construed as a potential conflict of interest.

## Abbreviations

TCA: tricarboxylic acid cycle
MeAIB: methylaminoisobutyrate
BCH: 2-aminobicyclo(2,2,1)heptane-2-carboxylic acid
SLC: Solute Carrier transporters
mTOR: mammalian target of rapamycin
FXR: farnesoid X receptor
RXR: 9-cis-retinoic acid receptor
IR-1: inverted repeat 1
EGF: epidermal growth factor
PKC: protein kinase C
MEK: tyrosine/threonine kinase
IGF: insulin-like growth factor
PI3K: phosphoinositide 3-kinase
SGK: serum/glucocorticoid regulated kinase
PKB: protein kinase B
MCT: lactate membrane transporter
ACE2: angiotensin-converting enzyme 2
APN: aminopeptidase N/CD13
CATs: cationic amino acid transporters
LATs: light subunits of amino acid transporters
HATs: heterodimeric amino acid transporters
TMS: trans-membrane domain
IPTG: isopropyl β-D-1-thiogalactopyranoside
DHT: dihydrotestosterone
ERK1/2: mitogen-activated protein kinase
APC: amino acid polyamine-organo cation family
EAAT1: excitatory amino acid transporter SLC1A3
EAAT2: excitatory amino acid transporter SLC1A2
PKA: protein kinase A
GLS1: glutaminase 1
OCTN: organic cation transporter novel
SERT: serotonine transporter
RMSD: Root Mean Square Deviation.
